# Supplementary material for: First direct evidence for direct cell-membrane penetrations of polycationic homopoly(amino acid)s produced by bacteria
Source: Commun Biol. 2022 Oct 26;5:1132. doi: 10.1038/s42003-022-04110-4 (PMC9606270; doi:10.1038/s42003-022-04110-4)
Supplement: Supplementary file 2 — Supplymentary information [file 42003_2022_4110_MOESM2_ESM.pdf]

Supplementary information

First direct evidence for direct cell-membrane penetrations of polycationic homopoly(amino acid)s produced by bacteria

Yamato Takeuchi[1], Kazunori Ushimaru[1,2], Kohei Kaneda[1], Chitose Maruyama[1,3,4], Takashi Ito[1,3], Kazuya Yamanaka[5], Yasushi Ogasawara[6], Hajime Katano[1], Yasuo Kato[7], Tohru Dairi[6], Yoshimitsu Hamano[1,3,4]*

[1] *Department of Bioscience, Fukui Prefectural University, 4-1-1 Matsuoka-Kenjojima, Yoshida-Gun, Fukui 910-1195, Japan.*

[2] *Research Institute for Sustainable Chemistry, National Institute of Advanced Industrial Science and Technology (AIST), Tsukuba, Ibaraki 305-8565, Japan*

[3] *Fukui Bioincubation Center (FBIC), Fukui Prefectural University, 4-1-1 Matsuoka-Kenjojima, Yoshida-Gun, Fukui 910-1195, Japan.*

[4] *MicrobeChem Inc., 4-1-1 Matsuoka-Kenjojima, Yoshida-Gun, Fukui 910-1195, Japan.*

[5] *Department of Life Science & Technology, Kansai University, 3-3-35 Yamate-cho, Suita, Osaka 564-8680, Japan.*

[6] *Graduate School of Engineering, Hokkaido University, N13-W8, Kita-ku, Sapporo, Hokkaido 060-8628, Japan.*

[7] *Department of Biotechnology, Toyama Prefectural University, 5180 Kurokawa, Imizu-shi, Toyama 939-0398, Japan.*

*e-mail: hamano@fpu.ac.jp

Contents of Supplementary Information

1. Supplementary Figures 1-7
2. Supplementary Tables 1-30
3. Supplementary Note

1. Supplementary Figures

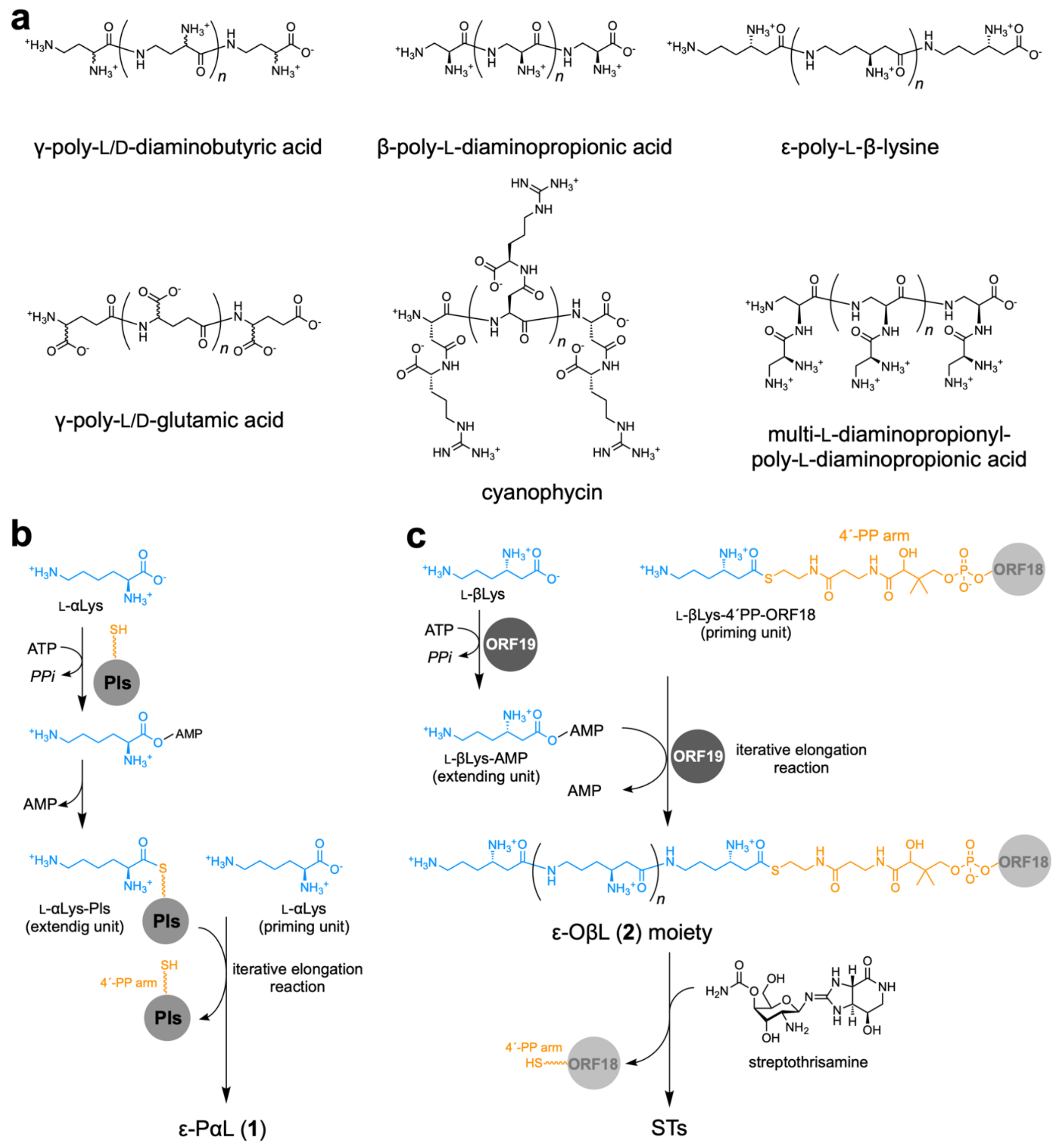

Supplementary Fig. 1 Biosynthetic mechanisms of polycationic lysine isopeptides.
a, Chemical structures of amino-acid homopolymers occurring in nature. **b**, Biosynthesis of ε-PαL (**1**). Polymer **1** is produced as a secondary metabolite by some *Streptomyces* strains. The biosynthetic enzyme (ε-PαL synthetase, Pls), a membrane-associated nonribosomal peptide synthetase (NRPS), polymerizes L-αLys to produce **1** with the peptide chain-length diversities (25–35 mer) by the iterative reactions. The L-αLys thioester form with the 4′-phosphopantetheine (4′-PP) arm of Pls is used as extending units during the polymerization reaction. **c**, Biosynthesis of ε-OβL (**2**) catalyzed by ORF19. In the ORF19

catalytic reaction, L-βLys covalently attached to the 4′-PP arm of ORF18 (NRPS) is used as a priming unit for the oligomerization reaction. ORF19 also mediates the ATP-dependent adenylation of L-βLys and uses the resulting L-βLys-AMP as the extending unit to grow the isopeptide chain of **2**. Notably, this chain elongation reaction iteratively occurs on the 4′-PP arm of ORF18; ORF19 never oligomerize directly with only L-βLys monomer units. Thus, ORF19 requires the L-βLys scaffold tethered to 4′PP-ORF18 as a priming unit.

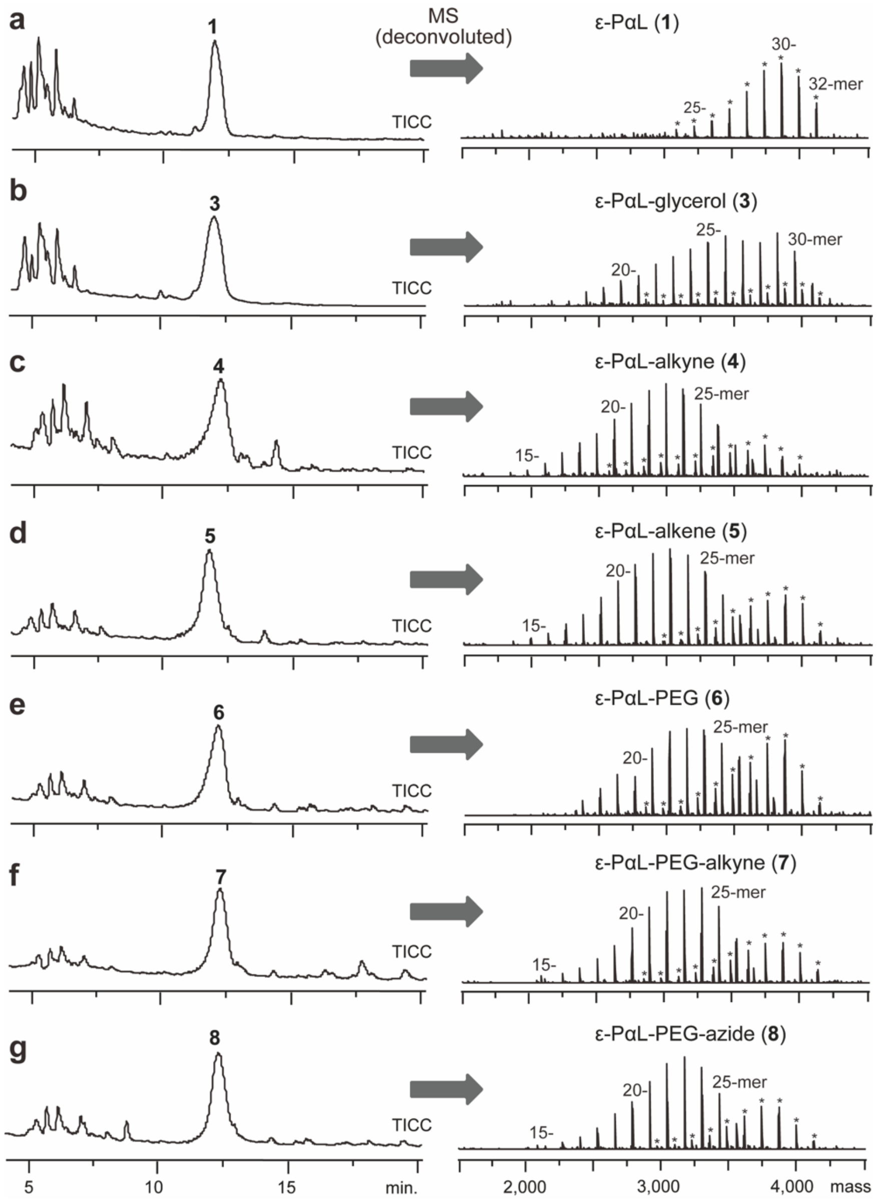

Supplementary Fig. 2 Production of ε-PαL ester derivatives *in vivo*.

a–g, HPLC-HR-ESI-MS analysis of the ε-PαL (**1**) ester derivatives produced by *Streptomyces albulus* NBRC14147. The NBRC14147 strain was cultivated in the culture media supplemented without (**a**) or with one of the following alcohols at 0.2% (w/v): glycerol (**b**), 2-propyn-1-ol (**c**), 3-buten-1-ol (**d**), polyethylene glycerol (PEG) (**e**), PEG-alkyne (**f**), or PEG-azide (**g**). Supernatants of the culture broths were analyzed by HPLC-HR-ESI-MS. The total ion current chromatograms (TICCs) are shown in the left panel. The deconvoluted mass spectra of **1** and the ε-PαL ester derivatives (**3**, **4**, **5**, **6**, **7**, and **8**) produced in the culture broth are shown in the right panel. The deconvoluted mass data are also summarized in **Supplementary Tables 1–7**. The productivities and abundance ratios of ε-PαL ester derivatives are summarized in **Supplementary Table 8**. Asterisks denote the deconvoluted mass spectra of **1** (unseparated by HPLC).

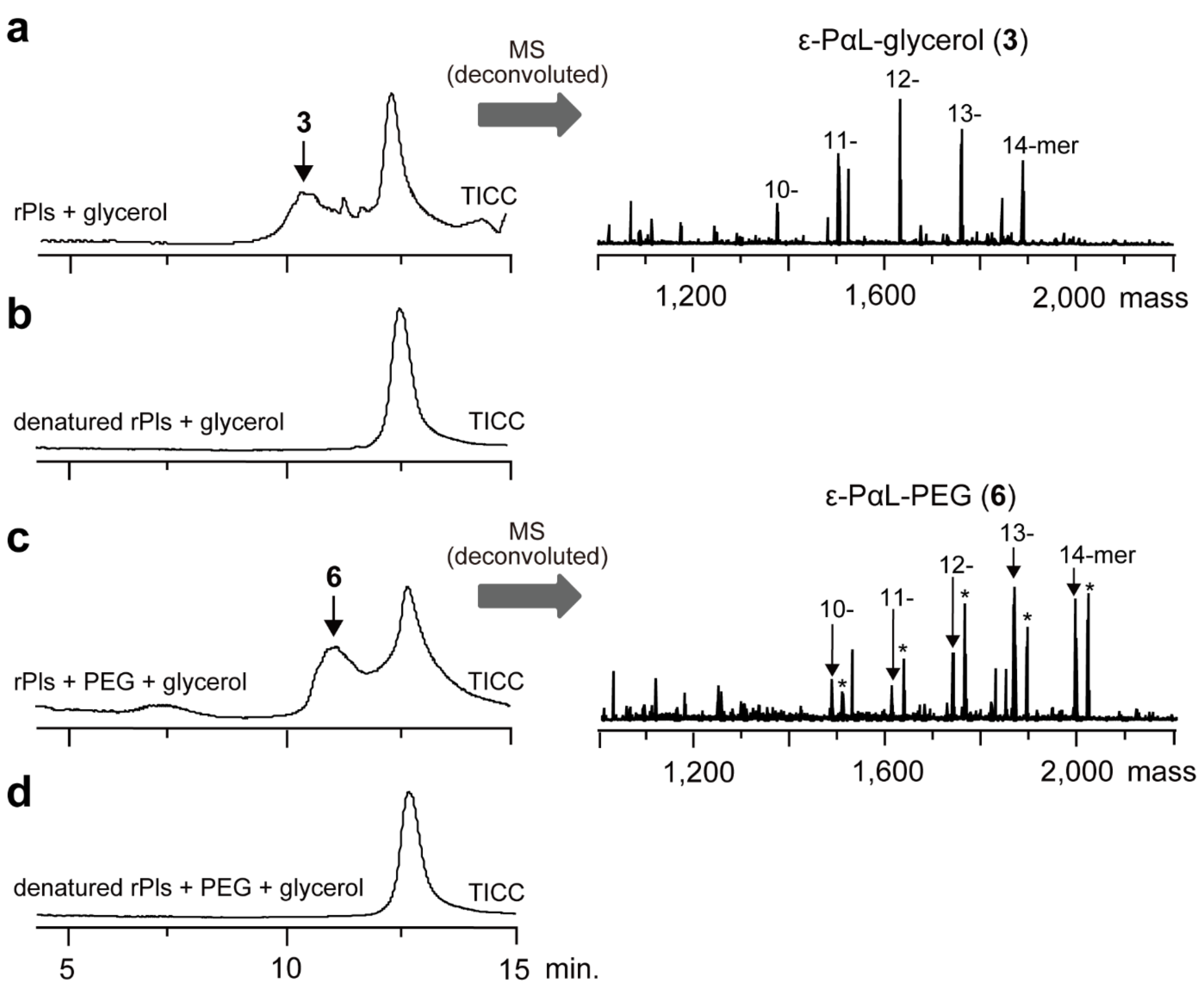

Supplementary Fig. 3 ε-PαL ester derivatives enzymatically synthesized by rPls.
a–d, HPLC-HR-ESI-MS analysis of the enzymatically synthesized ε-PαL-glycerol (**3**) and ε-PαL-PEG (**6**). The polymerization reactions by rPls (**a**, **c**) and the heat-denatured rPls (**b**, **d**) were performed, and then the reaction mixtures were analyzed by HPLC-HR-ESI-MS. Glycerol (20[w/v]%) was added into all reaction mixtures because rPls requires glycerol for its enzymatic activity *in vitro* (**a**–**d**). PEG was further added to the reaction mixture (**c**, **d**). The total ion current chromatograms (TICCs) are shown in the left panel. The deconvoluted mass spectra of **3** and **6**, which were enzymatically synthesized, are shown in the right panel. Asterisks denote the deconvoluted mass spectra of **3** (11–14-mer). The deconvoluted mass data are also summarized in **Supplementary Table 9, 10**.

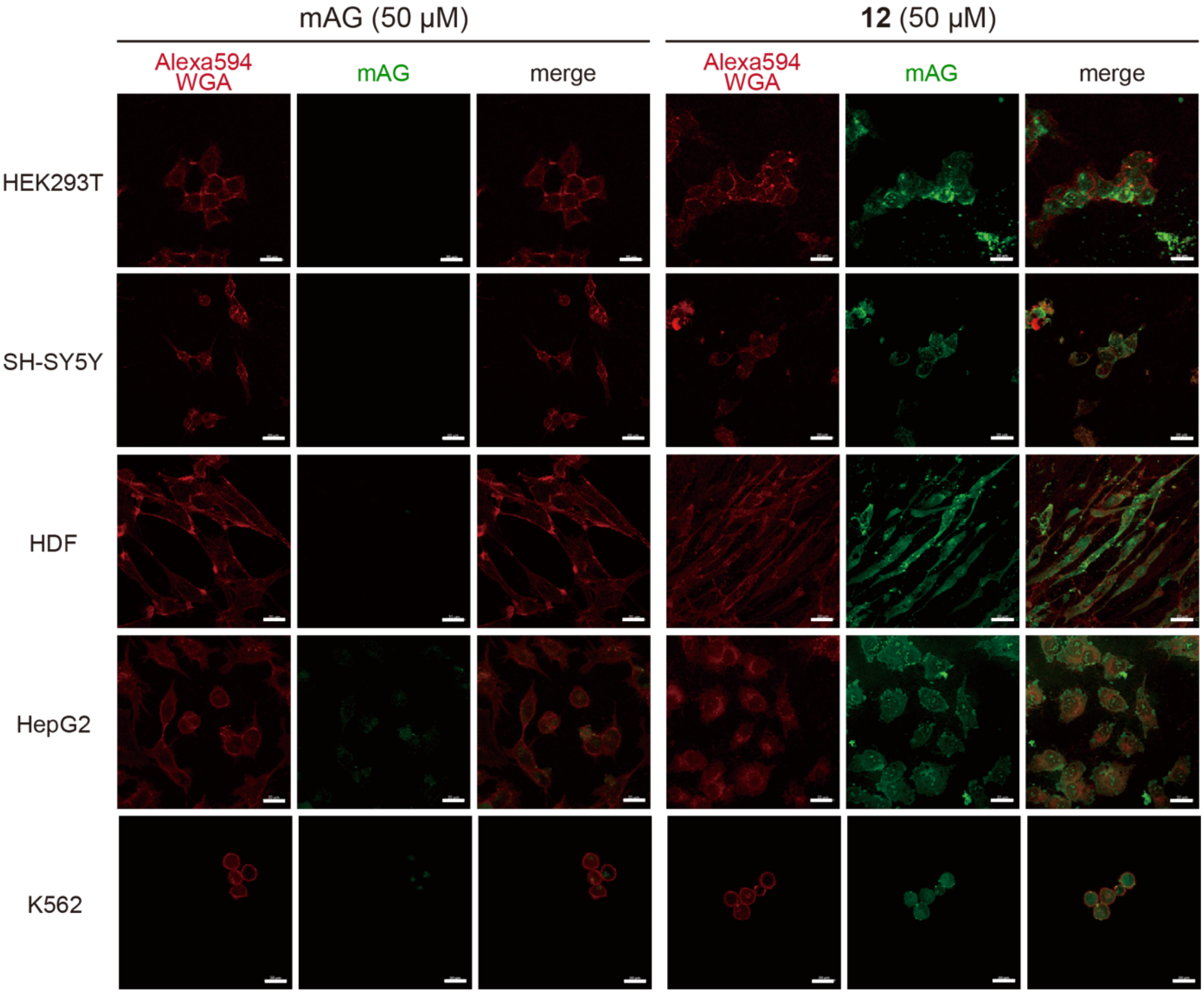

Supplementary Fig. 4 Cellular uptakes of ε-PαL-mAG (12).

mAG (50 μM) and **12** (50 μM) were incubated with HEK293T, SH-SY5Y, HDF, HepG2, and K562 cells for 60 min at 37°C under typical cell culture conditions with serum. The cellular localization (green) of **12** was determined by CM after washing and fixing the cells. Then, the cell membrane was stained with Alexa594-WGA (red). The representative CM images are shown. Scale bars, 20 μm.

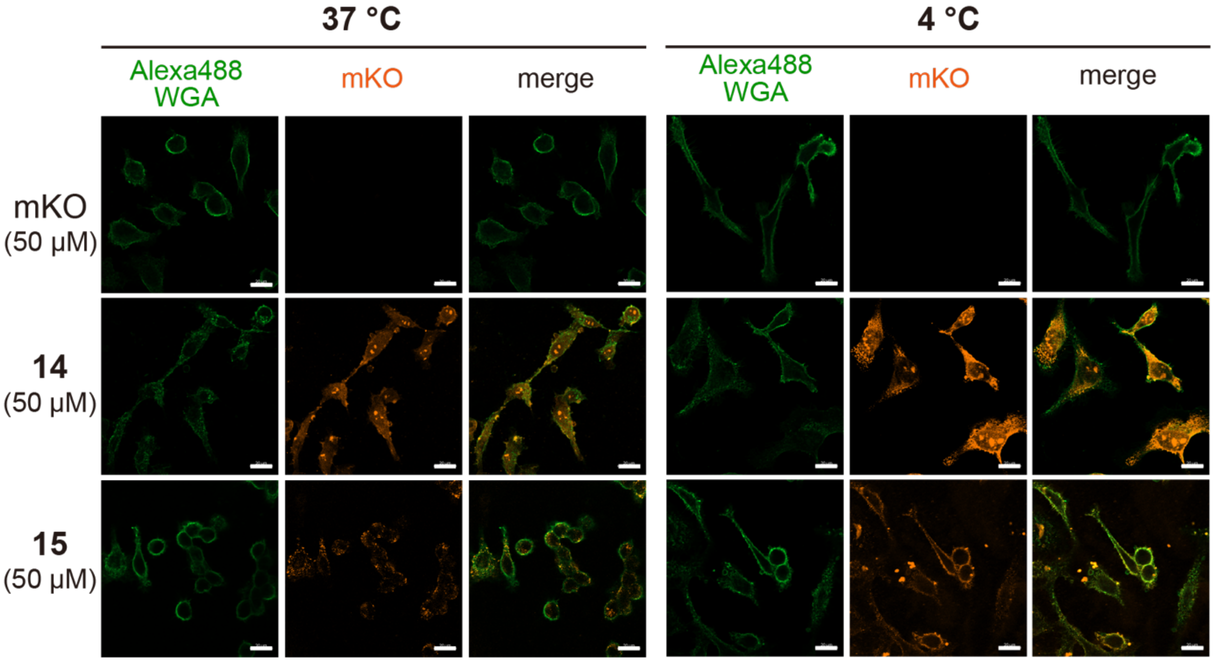

Supplementary Fig. 5 Cytosolic delivery of mKO conjugated with ε-PαL (1) or R8. HeLa cells were incubated with mKO (50 μM), ε-PαL-mKO (**14**) (50 μM), and R8-mKO (**15**) (50 μM) for 60 min at 37°C and 4°C under typical cell culture conditions with serum. The cellular localization (orange) of mKO, **14**, and **15** were determined by CM after washing and fixing the cells. Then, the cell membrane was stained with the membrane marker wheat germ agglutinin (Alexa488-WGA; green). The representative CM images are shown. Scale bars, 20 μm.

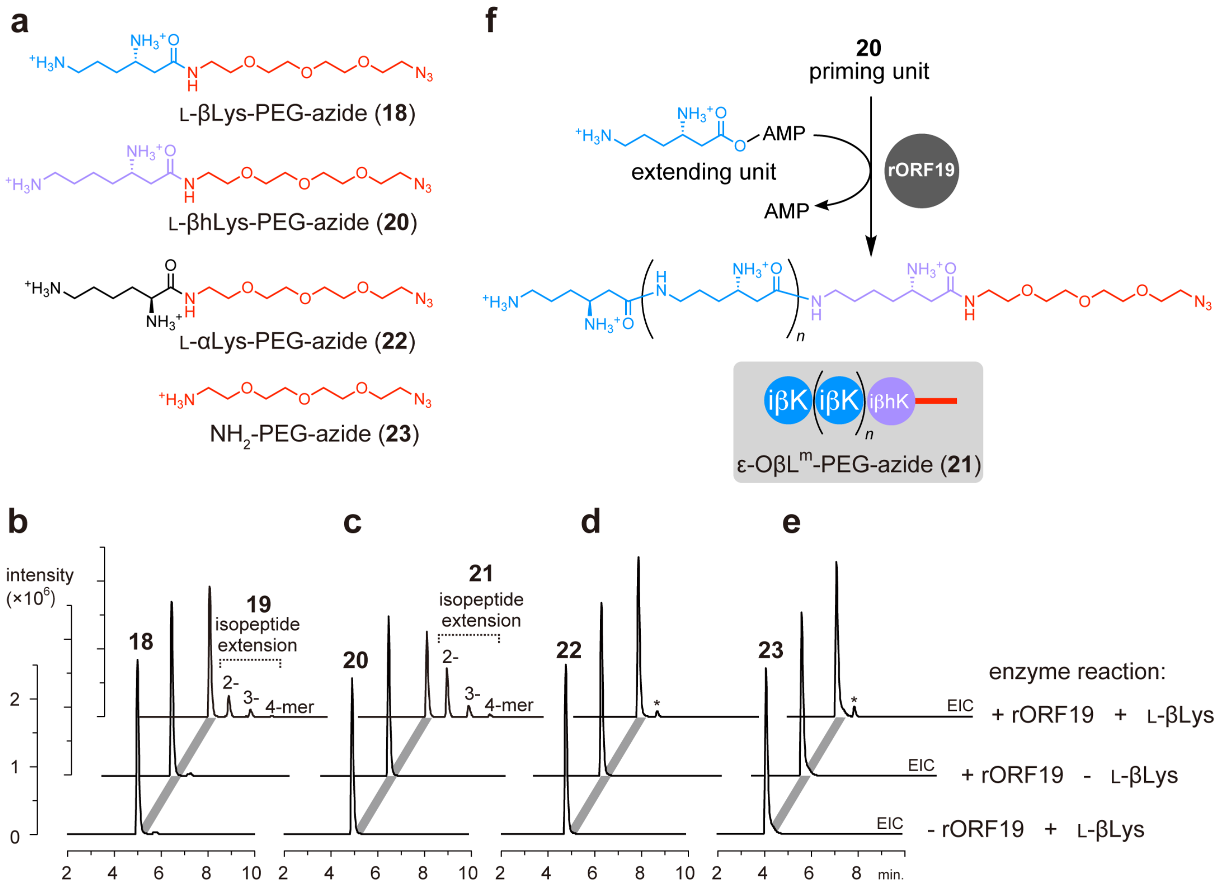

Supplementary Fig. 6 Chemoenzymatic synthesis of ε-OβL by rORF19.

a, The chemical structures of L-βLys-PEG-azide (**18**), L-βhLys-PEG-azide (**20**), L-αLys-PEG-azide (**22**), and NH_2-PEG-azide (**23**). These four priming units were employed for the rORF19 enzyme reactions. The priming units **18**, **20**, and **22** were chemically synthesized in the present study (**Supplementary Table 20**, **22**, **24**), although **23** was purchased. **b–e**, HPLC-HR-ESI-MS analysis of enzymatically synthesized ε-OβL. L-βLys oligomerization reactions by rORF19 were examined with the four priming units, **18** (**b**), **20** (**c**), **22** (**d**), and **23** (**e**). The enzyme reaction mixtures with or without rORF19 (0.1 mg/mL) and with or without L-βLys (extending unit) were analyzed by HPLC-HR-ESI-MS. The extracted ion chromatograms (EICs) are shown. The MS data of ε-OβL-PEG-azide (**19**), which was chemoenzymatically synthesized from **18** (**b**), are summarized in **Supplementary Table 21**. rORF19 was also able to accept **20** and produce ε-OβL^m-PEG-azide (**21**) (**c**). The MS data of **21** are summarized in **Supplementary Table 23**. The *m/z* 347.240 $[M+H]^+$ for L-αLys-PEG-azide (**22**) (calculated for $C_{14}H_{30}N_6O_4$, *m/z* 347.240 $[M+H]^+$) (**d**) and the *m/z* 219.145 $[M+H]^+$ for NH_2-PEG-azide (**23**) (calculated for $C_8H_{18}N_4O_3$, *m/z* 219.145 $[M+H]^+$) (**e**) were observed in the HPLC-HR-ESI-MS analysis. Asterisks denote L-β-lysyl-L-αLys-PEG-azide and L-β-lysyl-NH_2-PEG-azide, which might be non-enzymatically synthesized from L-β-lysyl-adenylate produced by rORF19. **f**, ε-OβL^m-PEG-azide (**21**) enzymatically synthesized by rORF19. iβK (light blue circles), isopeptide L-β-lysine monomer unit. iβhK (light purple circle), isopeptide L-β-homolysine monomer unit.

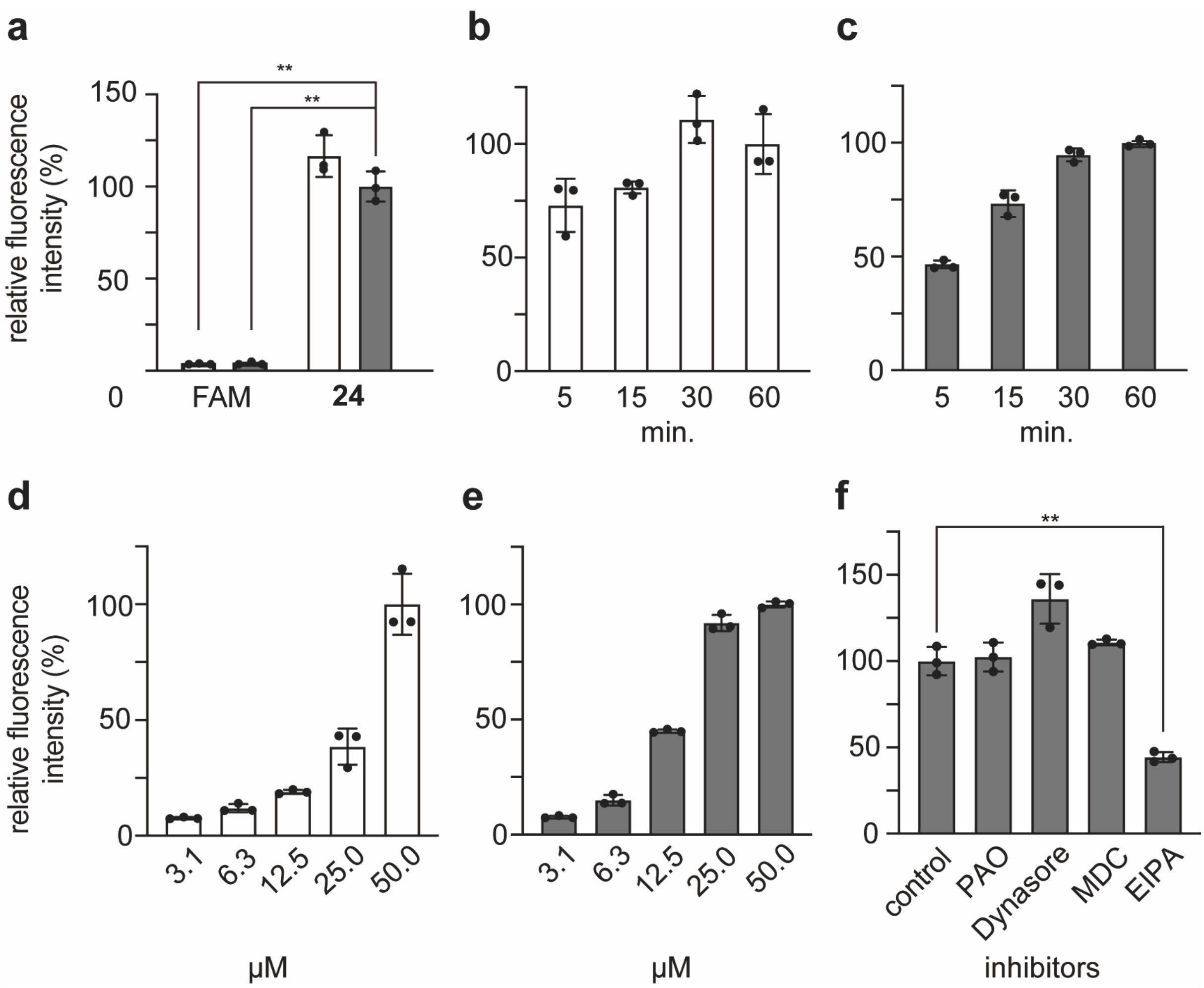

Supplementary Fig. 7 Cellular uptakes of ε-OβL^{m}-FAM (24).

a–f, Relative cellular uptakes of ε-OβL^{m}-FAM (**24**) consisting of 4–13 mer at 37°C (light gray) and 4°C (white) in HeLa cells. FAM (50 μM) and **24** (50 μM) were incubated with cells for 60 min at 37°C and 4°C under a typical cell culture condition with serum (**a**). The results are presented as the mean±s.d. (n=3). **Significant at p<0.01 by two-way ANOVA followed by Sidak's multiple comparisons test. Compound **24** (50 μM) was incubated with cells for 5–60 min at 4°C (**b**) and 37°C (**c**). Compound **24** (3.1–50 μM) was incubated with cells for 60 min at 4°C (**d**) and 37°C (**e**). Compound **24** (50 μM) was incubated with cells for 60 min at 37°C in the culture medium supplemented with an endocytosis/macropinocytosis inhibitor (PAO, Dynasore, MDC, or EIPA) (**f**). The results are presented as the mean±s.d. (n=3). **Significant at *p*<0.01 by one-way ANOVA followed by Tukey's multiple comparisons test.

2. Supplementary tables

Supplementary Table 1

The mass data of ε-PαL (**1**) produced by *S. albulus* NBRC14147

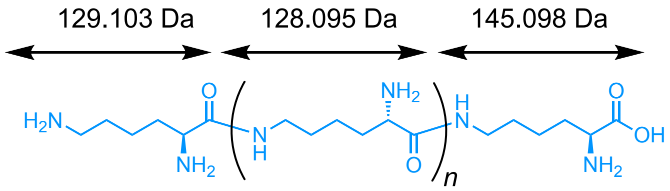

ε-PαL (**1**)

compound (**ID**)	peptide -chain length	molecular formula	calculated mass [a]	observed mass (deconvoluted) [a]	monomer-unit difference [b] (Da)
**1**	24 mer	$C_{144}H_{290}N_{48}O_{25}$	3,092.289	3,092.280	Δ 128.093
	25 mer	$C_{150}H_{302}N_{50}O_{26}$	3,220.384	3,220.373	Δ 128.088
	26 mer	$C_{156}H_{314}N_{52}O_{27}$	3,348.479	3,348.461	Δ 128.105
	27 mer	$C_{162}H_{326}N_{54}O_{28}$	3,476.574	3,476.566	Δ 128.105
	28 mer	$C_{168}H_{338}N_{56}O_{29}$	3,604.669	3,604.645	Δ 128.079
	29 mer	$C_{174}H_{350}N_{58}O_{30}$	3,732.764	3,732.746	Δ 128.098
	30 mer	$C_{180}H_{362}N_{60}O_{31}$	3,860.859	3,860.844	Δ 128.094
	31 mer	$C_{186}H_{374}N_{62}O_{32}$	3,988.954	3,988.938	Δ 128.092
	32 mer	$C_{192}H_{386}N_{64}O_{33}$	4,117.049	4,117.030	

[a] The values are shown for the monoisotopic masses.
[b] The molecular mass of the L-αLys monomer unit is calculated to be 128.095 Da.

Supplementary Table 2

The mass data of ε-PαL-glycerol (**3**) produced by *S. albulus* NBRC14147

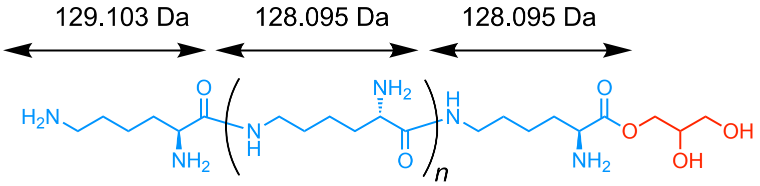

ε-PαL-glycerol (**3**)

compound (**ID**)	peptide -chain length	molecular formula	calculated mass [a]	observed mass (deconvoluted) [a]	monomer-unit difference [b] (Da)
**3**	16 mer	$C_{99}H_{200}N_{32}O_{19}$	2,141.566	2,141.567	Δ 128.095
	17 mer	$C_{105}H_{212}N_{34}O_{20}$	2,269.661	2,269.662	Δ 128.095
	18 mer	$C_{111}H_{224}N_{36}O_{21}$	2,397.756	2,397.757	Δ 128.096
	19 mer	$C_{117}H_{236}N_{38}O_{22}$	2,525.851	2,525.853	Δ 128.084
	20 mer	$C_{123}H_{248}N_{40}O_{23}$	2,653.946	2,653.937	Δ 128.111
	21 mer	$C_{129}H_{260}N_{42}O_{24}$	2,782.041	2,782.048	Δ 128.087
	22 mer	$C_{135}H_{272}N_{44}O_{25}$	2,910.136	2,910.135	Δ 128.102
	23 mer	$C_{141}H_{284}N_{46}O_{26}$	3,038.231	3,038.237	Δ 128.093
	24 mer	$C_{147}H_{296}N_{48}O_{27}$	3,166.326	3,166.330	Δ 128.097
	25 mer	$C_{153}H_{308}N_{50}O_{28}$	3,294.421	3,294.427	Δ 128.093
	26 mer	$C_{159}H_{320}N_{52}O_{29}$	3,422.516	3,422.520	Δ 128.090
	27 mer	$C_{165}H_{332}N_{54}O_{30}$	3,550.611	3,550.610	Δ 128.099
	28 mer	$C_{171}H_{344}N_{56}O_{31}$	3,678.706	3,678.709	Δ 128.092
	29 mer	$C_{177}H_{356}N_{58}O_{32}$	3,806.801	3,806.801	Δ 128.098
	30 mer	$C_{183}H_{368}N_{60}O_{33}$	3,934.896	3,934.899	Δ 128.094
	31 mer	$C_{189}H_{380}N_{62}O_{34}$	4,062.991	4,062.993	Δ 128.092
	32 mer	$C_{195}H_{392}N_{64}O_{35}$	4,191.086	4,191.085	

[a] The values are shown for the monoisotopic masses.
[b] The molecular mass of the L-αLys monomer unit is calculated to be 128.095 Da.

Supplementary Table 3

The mass data of ε-PαL-alkyne (**4**) produced by *S. albulus* NBRC14147

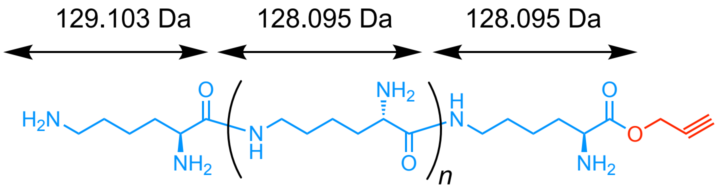

ε-PαL-alkyne (**4**)

compound (**ID**)	peptide -chain length	molecular formula	calculated mass [a]	observed mass (deconvoluted) [a]	monomer-unit difference [b] (Da)
**4**	14 mer	$C_{87}H_{172}N_{28}O_{15}$	1,849.355	1,849.354	Δ 128.099
	15 mer	$C_{93}H_{184}N_{30}O_{16}$	1,977.450	1,977.453	Δ 128.095
	16 mer	$C_{99}H_{196}N_{32}O_{17}$	2,105.545	2,105.548	Δ 128.092
	17 mer	$C_{105}H_{208}N_{34}O_{18}$	2,233.640	2,233.640	Δ 128.098
	18 mer	$C_{111}H_{220}N_{36}O_{19}$	2,361.735	2,361.738	Δ 128.095
	19 mer	$C_{117}H_{232}N_{38}O_{20}$	2,489.830	2,489.833	Δ 128.095
	20 mer	$C_{123}H_{244}N_{40}O_{21}$	2,617.925	2,617.928	Δ 128.103
	21 mer	$C_{129}H_{256}N_{42}O_{22}$	2,746.020	2,746.031	Δ 128.087
	22 mer	$C_{135}H_{268}N_{44}O_{23}$	2,874.115	2,874.118	Δ 128.097
	23 mer	$C_{141}H_{280}N_{46}O_{24}$	3,002.210	3,002.215	Δ 128.091
	24 mer	$C_{147}H_{292}N_{48}O_{25}$	3,130.305	3,130.306	Δ 128.097
	25 mer	$C_{153}H_{304}N_{50}O_{26}$	3,258.400	3,258.403	Δ 128.097
	26 mer	$C_{159}H_{316}N_{52}O_{27}$	3,386.495	3,386.500	Δ 128.074
	27 mer	$C_{165}H_{328}N_{54}O_{28}$	3,514.590	3,514.574	Δ 128.102
	28 mer	$C_{171}H_{340}N_{56}O_{29}$	3,642.685	3,642.676	

[a] The values are shown for the monoisotopic masses.
[b] The molecular mass of the L-αLys monomer unit is calculated to be 128.095 Da.

Supplementary Table 4

The mass data of ε-PαL-alkene (**5**) produced by *S. albulus* NBRC14147

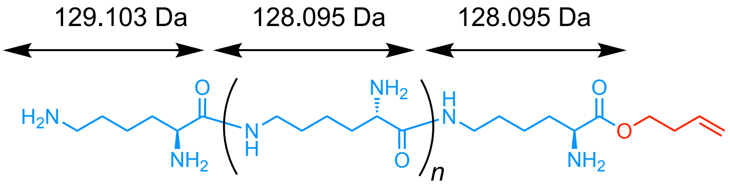

ε-PαL-alkene (**5**)

compound (**ID**)	peptide -chain length	molecular formula	calculated mass [a]	observed mass (deconvoluted) [a]	monomer-unit difference [b] (Da)
**5**	14 mer	$C_{88}H_{176}N_{28}O_{15}$	1,865.386	1,865.373	Δ 128.102
	15 mer	$C_{94}H_{182}N_{30}O_{16}$	1,993.481	1,993.475	Δ 128.079
	16 mer	$C_{100}H_{200}N_{32}O_{17}$	2,121.576	2,121.554	Δ 128.112
	17 mer	$C_{106}H_{212}N_{34}O_{18}$	2,249.671	2,249.666	Δ 128.099
	18 mer	$C_{112}H_{224}N_{36}O_{19}$	2,377.766	2,377.765	Δ 128.097
	19 mer	$C_{118}H_{236}N_{38}O_{20}$	2,505.861	2,505.862	Δ 128.093
	20 mer	$C_{124}H_{248}N_{40}O_{21}$	2,633.956	2,633.955	Δ 128.099
	21 mer	$C_{130}H_{260}N_{42}O_{22}$	2,762.051	2,762.054	Δ 128.092
	22 mer	$C_{136}H_{272}N_{44}O_{23}$	2,890.146	2,890.146	Δ 128.096
	23 mer	$C_{142}H_{284}N_{46}O_{24}$	3,018.241	3,018.242	Δ 128.100
	24 mer	$C_{148}H_{296}N_{48}O_{25}$	3,146.336	3,146.342	Δ 128.093
	25 mer	$C_{154}H_{308}N_{50}O_{26}$	3,274.431	3,274.435	Δ 128.095
	26 mer	$C_{160}H_{320}N_{52}O_{27}$	3,402.526	3,402.530	Δ 128.095
	27 mer	$C_{166}H_{332}N_{54}O_{28}$	3,530.621	3,530.625	Δ 128.091
	28 mer	$C_{172}H_{344}N_{56}O_{29}$	3,658.716	3,658.716	

[a] The values are shown for the monoisotopic masses.

[b] The molecular mass of the L-αLys monomer unit is calculated to be 128.095 Da.

Supplementary Table 5

The mass data of ε-PαL-PEG (**6**) produced by *S. albulus* NBRC14147

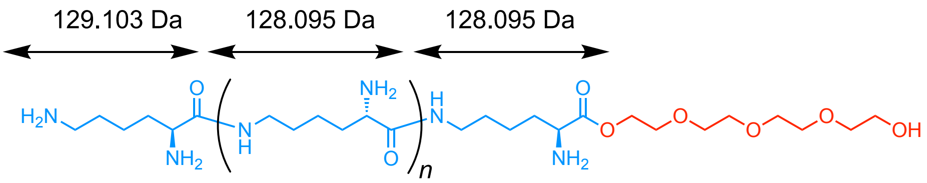

ε-PαL-PEG (**6**)

compound (**ID**)	peptide -chain length	molecular formula	calculated mass [a]	observed mass (deconvoluted) [a]	monomer-unit difference [b] (Da)
**6**	17 mer	$C_{110}H_{222}N_{34}O_{22}$	2,371.729	2,371.732	Δ 128.093
	18 mer	$C_{116}H_{234}N_{36}O_{23}$	2,499.824	2,499.825	Δ 128.097
	19 mer	$C_{122}H_{246}N_{38}O_{24}$	2,627.919	2,627.922	Δ 128.097
	20 mer	$C_{128}H_{258}N_{40}O_{25}$	2,756.014	2,756.019	Δ 128.087
	21 mer	$C_{134}H_{270}N_{42}O_{26}$	2,884.109	2,884.106	Δ 128.101
	22 mer	$C_{140}H_{282}N_{44}O_{27}$	3,012.204	3,012.207	Δ 128.097
	23 mer	$C_{146}H_{294}N_{46}O_{28}$	3,140.299	3,140.304	Δ 128.097
	24 mer	$C_{152}H_{306}N_{48}O_{29}$	3,268.394	3,268.401	Δ 128.095
	25 mer	$C_{158}H_{318}N_{50}O_{30}$	3,396.489	3,396.496	Δ 128.091
	26 mer	$C_{164}H_{330}N_{52}O_{31}$	3,524.584	3,524.587	Δ 128.092
	27 mer	$C_{170}H_{342}N_{54}O_{32}$	3,652.679	3,652.679	Δ 128.080
	28 mer	$C_{176}H_{354}N_{56}O_{33}$	3,780.774	3,780.759	

[a] The values are shown for the monoisotopic masses.

[b] The molecular mass of the L-αLys monomer unit is calculated to be 128.095 Da.

Supplementary Table 6

The mass data of ε-PαL-PEG-alkyne (**7**) produced by *S. albulus* NBRC14147

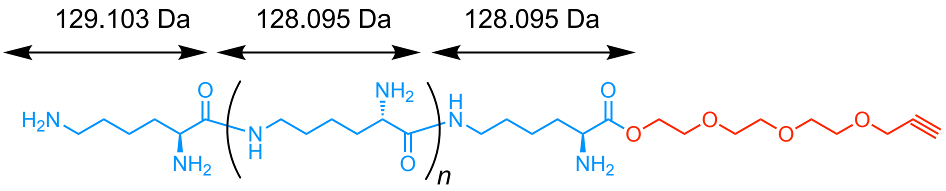

ε-PαL-PEG-alkyne (**7**)

compound (**ID**)	peptide -chain length	molecular formula	calculated mass [a]	observed mass (deconvoluted) [a]	monomer-unit difference [b] (Da)
**7**	15 mer	$C_{99}H_{196}N_{30}O_{19}$	2,109.529	2,109.531	Δ 128.095
	16 mer	$C_{105}H_{208}N_{32}O_{20}$	2,237.624	2,237.626	Δ 128.095
	17 mer	$C_{111}H_{220}N_{34}O_{21}$	2,365.719	2,365.721	Δ 128.090
	18 mer	$C_{117}H_{232}N_{36}O_{22}$	2,493.814	2,493.811	Δ 128.101
	19 mer	$C_{123}H_{244}N_{38}O_{23}$	2,621.909	2,621.912	Δ 128.096
	20 mer	$C_{129}H_{256}N_{40}O_{24}$	2,750.004	2,750.008	Δ 128.094
	21 mer	$C_{135}H_{268}N_{42}O_{25}$	2,878.099	2,878.102	Δ 128.093
	22 mer	$C_{141}H_{280}N_{44}O_{26}$	3,006.194	3,006.195	Δ 128.093
	23 mer	$C_{147}H_{292}N_{46}O_{27}$	3,134.289	3,134.288	Δ 128.096
	24 mer	$C_{153}H_{304}N_{48}O_{28}$	3,262.383	3,262.384	Δ 128.100
	25 mer	$C_{159}H_{316}N_{50}O_{29}$	3,390.478	3,390.484	Δ 128.092
	26 mer	$C_{165}H_{328}N_{52}O_{30}$	3,518.573	3,518.576	Δ 128.086
	27 mer	$C_{171}H_{340}N_{54}O_{31}$	3,646.668	3,646.662	

[a] The values are shown for the monoisotopic masses.
[b] The molecular mass of the L-αLys monomer unit is calculated to be 128.095 Da.

Supplementary Table 7

The mass data of ε-PαL-PEG-azide (**8**) produced by *S. albulus* NBRC14147

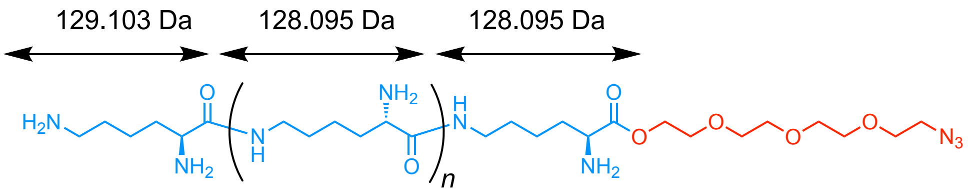

ε-PαL-PEG-azide (**8**)

compound (**ID**)	peptide -chain length	molecular formula	calculated mass [a]	observed mass (deconvoluted) [a]	monomer-unit difference [b] (Da)
**8**	16 mer	$C_{104}H_{209}N_{35}O_{20}$	2,268.641	2,268.656	
					Δ 128.095
	17 mer	$C_{110}H_{221}N_{37}O_{21}$	2,396.736	2,396.748	
					Δ 128.084
	18 mer	$C_{116}H_{233}N_{39}O_{22}$	2,524.831	2,524.832	
					Δ 128.107
	19 mer	$C_{122}H_{245}N_{41}O_{23}$	2,652.926	2,652.939	
					Δ 128.063
	20 mer	$C_{128}H_{257}N_{43}O_{24}$	2,781.021	2,781.022	
					Δ 128.115
	21 mer	$C_{134}H_{269}N_{45}O_{25}$	2,909.116	2,909.117	
					Δ 128.099
	22 mer	$C_{140}H_{281}N_{47}O_{26}$	3,037.211	3,037.216	
					Δ 128.094
	23 mer	$C_{146}H_{293}N_{49}O_{27}$	3,165.306	3,165.310	
					Δ 128.099
	24 mer	$C_{152}H_{305}N_{51}O_{28}$	3,293.401	3,293.409	
					Δ 128.091
	25 mer	$C_{158}H_{317}N_{53}O_{29}$	3,421.495	3,421.500	
					Δ 128.090
	26 mer	$C_{164}H_{329}N_{55}O_{30}$	3,549.590	3,549.590	

[a] The values are shown for the monoisotopic masses.
[b] The molecular mass of the L-αLys monomer unit is calculated to be 128.095 Da.

Supplementary Table 8

Productivities and abundance ratios of ε-PαL ester derivatives produced by *S. albulus* NBRC14147

alcohols supplemented to culture medium	total productivity of ε-PαL plus ε-PαL ester derivative (mg/L)	ε-PαL ester derivative abundance ratio (%) [a]
2-propyn-1-ol 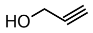	106.4 ± 7.3	75.6 ± 3.3
3-buten-1-ol 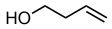	109.7 ± 1.6	70.5 ± 0.3
polyethylene glycerol (PEG) 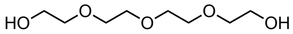	109.8 ± 0.4	58.1 ± 0.4
PEG-alkyne 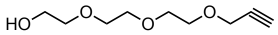	109.4 ± 1.4	72.3 ± 1.2
PEG-azide 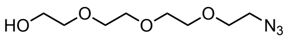	107.6 ± 2.9	71.0 ± 2.9

[a] The values were calculated based on the peak area of deconvoluted mass spectra. Each value represents the mean ± SD of triplicate experiments.

Supplementary Table 9

The mass data of ε-PαL-glycerol (**3**) enzymatically synthesized by rPls

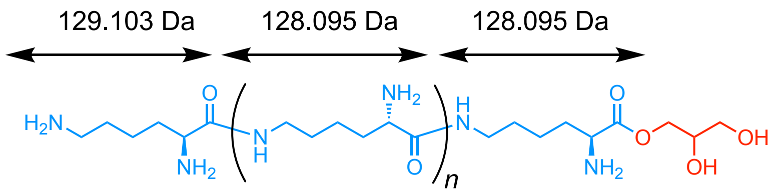

ε-PαL-glycerol (**3**)

compound (**ID**)	peptide -chain length	molecular formula	calculated mass [a]	observed mass (deconvoluted) [a]	monomer-unit difference [b] (Da)
**3**	10 mer	$C_{63}H_{128}N_{20}O_{13}$	1,372.996	1,372.996	Δ 128.096
	11 mer	$C_{69}H_{140}N_{22}O_{14}$	1,501.092	1,501.092	Δ 128.096
	12 mer	$C_{75}H_{152}N_{24}O_{15}$	1,629.186	1,629.188	Δ 128.095
	13 mer	$C_{81}H_{164}N_{26}O_{16}$	1,757.281	1,757.283	Δ 128.094
	14 mer	$C_{87}H_{176}N_{28}O_{17}$	1,885.376	1,885.377	

[a] The values are shown for the monoisotopic masses.
[b] The molecular mass of the L-αLys monomer unit is calculated to be 128.095 Da.

Supplementary Table 10

The mass data of ε-PαL-PEG (**6**) enzymatically synthesized by rPIs

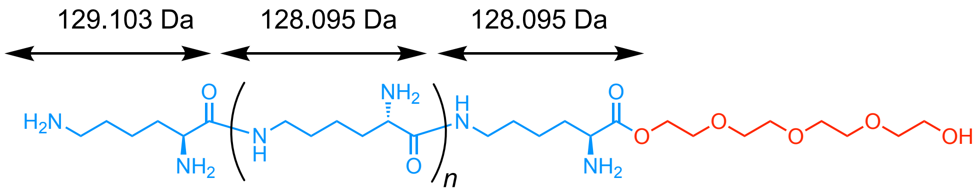

ε-PαL-PEG (**6**)

compound (**ID**)	peptide -chain length	molecular formula	calculated mass [a]	observed mass (deconvoluted) [a]	monomer-unit difference [b] (Da)
**6**	10 mer	$C_{68}H_{138}N_{20}O_{15}$	1,475.065	1,475.064	Δ 128.098
	11 mer	$C_{74}H_{150}N_{22}O_{16}$	1,603.160	1,603.162	Δ 128.092
	12 mer	$C_{80}H_{162}N_{24}O_{17}$	1,731.254	1,731.254	Δ 128.096
	13 mer	$C_{86}H_{174}N_{26}O_{18}$	1,859.349	1,859.350	Δ 128.092
	14 mer	$C_{92}H_{186}N_{28}O_{19}$	1,987.444	1,987.442	

[a] The values are shown for the monoisotopic masses.
[b] The molecular mass of the L-αLys monomer unit is calculated to be 128.095 Da.

Supplementary Table 11

Elucidation of the chemical structure of ε-PαL-PEG-azide (**8**) by NMR

^{13}C (150 MHz) and ^{1}H (600 MHz) NMR data[a] for ε-PαL-PEG-azide (**8**)

No.	δ_C	δ_H (multiplicity, J = Hz)
1	50.2	3.31(t, J = 5.6)
2	69.6	3.53 (m)
3	69.6	3.53 (m)
4	69.6	3.53 (m)
5	69.6	3.53 (m)
6	69.6	3.53 (m)
7	68.2	3.64 (dd, J = 5.7, 3.3)
8	65.4	4.24 (dd, J = 6.2)
9	169.6	
10	53.3	3.74 (t, J = 6.8)
11	30.5	1.68 (m)
12	21.7	1.20 (m)
13	27.9	1.39 (m)
14	39.1	3.04 (m)
15	169.6	
16	53.1	3.99 (t, J = 6.5)
17	29.4	1.79 (m)
18	21.4	1.26 (m)
19	26.4	1.52 (m)
20	39.0	2.81(t, J = 7.9)

[a] NMR spectra were obtained with the JNM-ECA600 in D_2O. Water signal was used as an internal standard (δ_H 4.79 ppm).

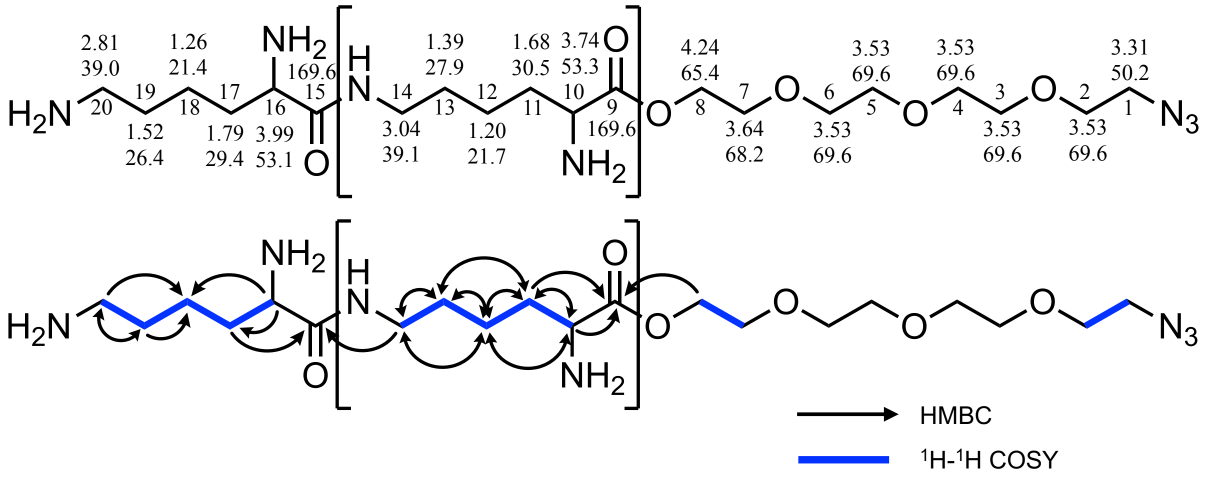

Supplementary Table 12

The HPLC-HR-ESI-MS data of ε-PαL-FAM (**10**) chemically synthesized

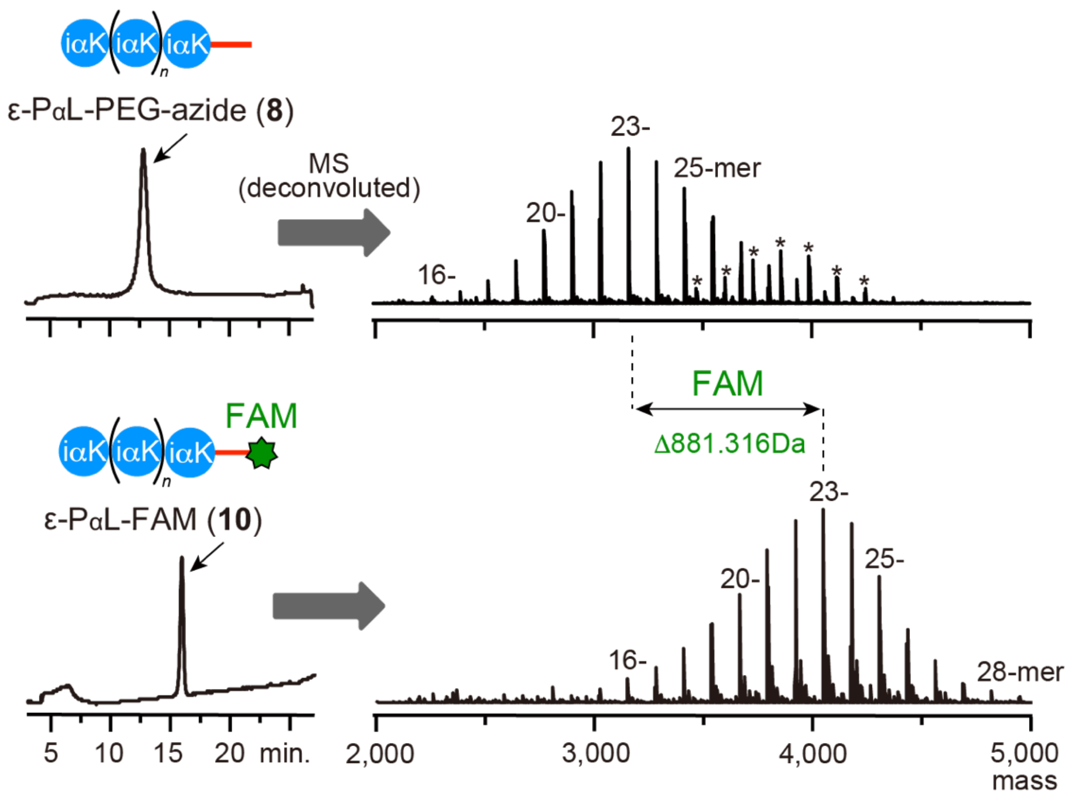

Asterisks denote the deconvoluted mass spectra of **1** (unseparated by HPLC). The total ion current (TIC) chromatograms (Right) and the deconvoluted MS spectra (Right) are shown.

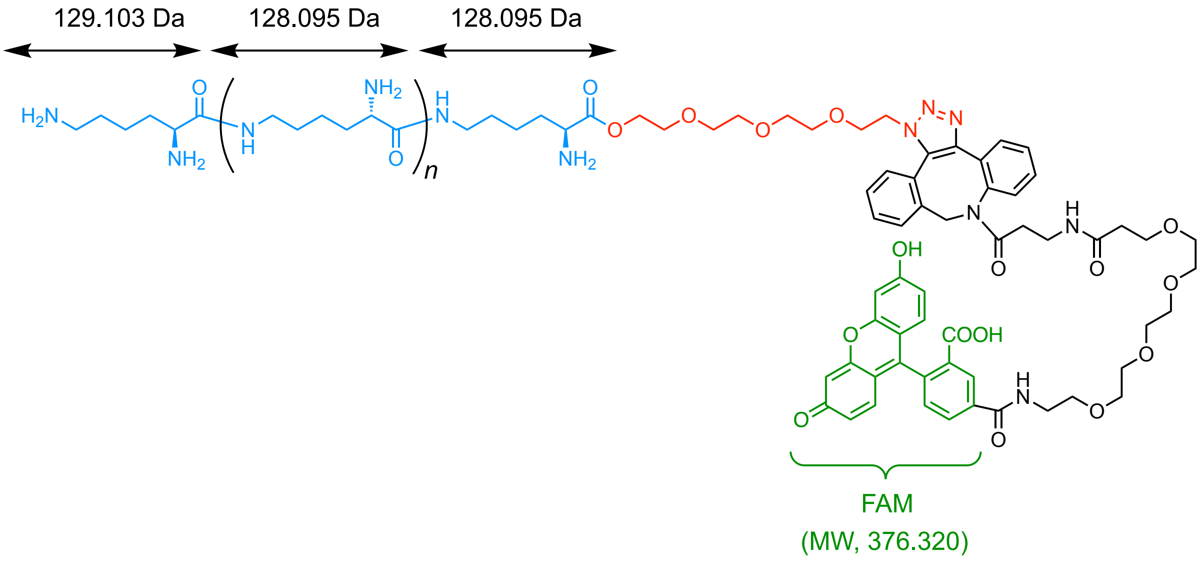

(Continued on the next page)

compound (**ID**)	peptide -chain length	molecular formula	calculated mass [a]	observed mass (deconvoluted) [a]	monomer-unit difference [b] (Da)
**10**	16 mer	$C_{154}H_{256}N_{38}O_{32}$	3,149.957	3,149.932	Δ 128.124
	17 mer	$C_{160}H_{268}N_{40}O_{33}$	3,278.052	3,278.056	Δ 128.106
	18 mer	$C_{166}H_{280}N_{42}O_{34}$	3,406.147	3,406.162	Δ 128.082
	19 mer	$C_{172}H_{292}N_{44}O_{35}$	3,534.242	3,534.244	Δ 128.090
	20 mer	$C_{178}H_{304}N_{46}O_{36}$	3,662.337	3,662.334	Δ 128.100
	21 mer	$C_{184}H_{316}N_{48}O_{37}$	3,790.432	3,790.434	Δ 128.095
	22 mer	$C_{190}H_{328}N_{50}O_{38}$	3,918.527	3,918.529	Δ 128.095
	23 mer	$C_{196}H_{340}N_{52}O_{39}$	4,046.621	4,046.623	Δ 128.094
	24 mer	$C_{202}H_{352}N_{54}O_{40}$	4,174.716	4,174.720	Δ 128.096
	25 mer	$C_{208}H_{364}N_{56}O_{41}$	4,302.811	4,302.816	Δ 128.091
	26 mer	$C_{214}H_{376}N_{58}O_{42}$	4,430.906	4,430.907	Δ 128.111
	27 mer	$C_{220}H_{388}N_{60}O_{43}$	4,559.001	4,559.018	Δ 128.097
	28 mer	$C_{226}H_{400}N_{62}O_{44}$	4,687.096	4,687.115	

[a] The values are shown for the monoisotopic masses.
[b] The molecular mass of the L-αLys monomer unit is calculated to be 128.095 Da.

Supplementary Table 13

The HPLC-HR-ESI-MS data of ε-PαL-mAG (**12**) chemically synthesized

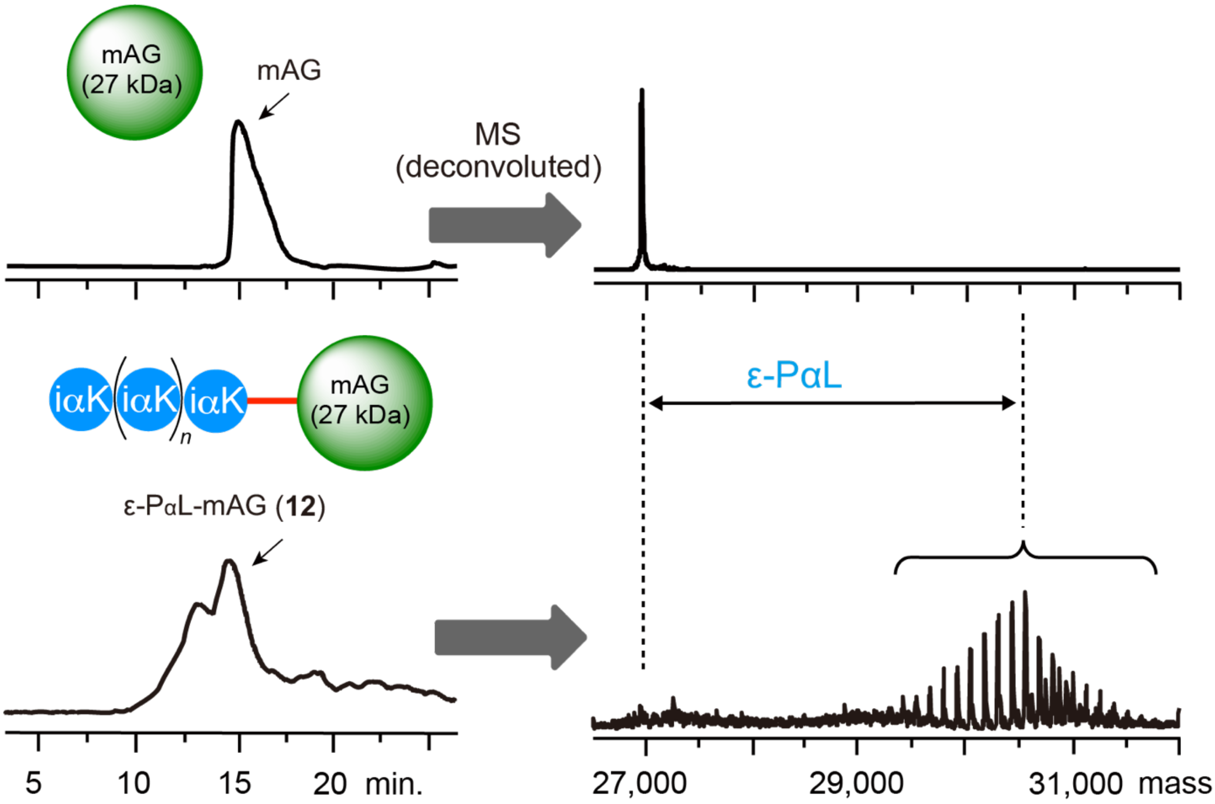

The total ion current (TIC) chromatograms (Right) and the deconvoluted MS spectra (Right) are shown.

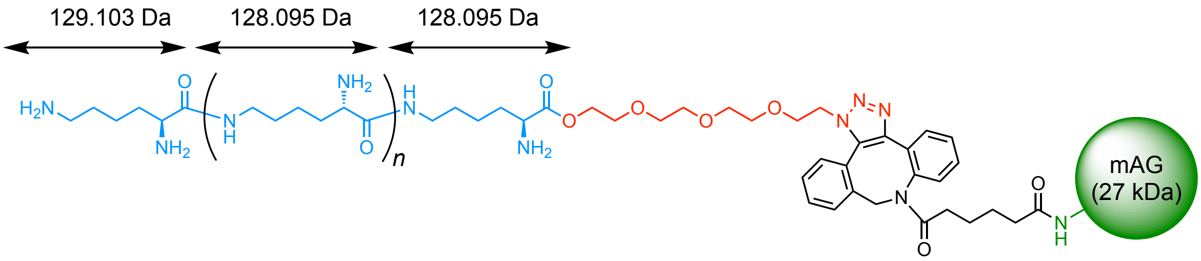

(Continued on the next page)

compound (**ID**)	peptide -chain length	molecular formula	calculated mass [a]	observed mass (deconvoluted) [a]	monomer-unit difference [b] (Da)	mass discrepancy [c] (Da)
mAG	N/A	$C_{1210}H_{1846}N_{328}O_{350}S_{12}$	26,972.42	26,951.97	N/A	-20.45
**12**	17 mer	$C_{1341}H_{2084}N_{366}O_{373}S_{12}$	29,685.95	29,664.62	Δ 128.90	-21.33
	18 mer	$C_{1347}H_{2096}N_{368}O_{374}S_{12}$	29,814.13	29,793.52	Δ 128.22	-20.61
	19 mer	$C_{1353}H_{2108}N_{370}O_{375}S_{12}$	29,942.30	29,921.74	Δ 128.60	-20.56
	20 mer	$C_{1359}H_{2120}N_{372}O_{376}S_{12}$	30,070.47	30,050.34	Δ 128.00	-20.13
	21 mer	$C_{1365}H_{2132}N_{374}O_{377}S_{12}$	30,198.64	30,178.34	Δ 127.67	-20.30
	22 mer	$C_{1371}H_{2144}N_{376}O_{378}S_{12}$	30,326.82	30,306.01	Δ 128.92	-20.81
	23 mer	$C_{1377}H_{2156}N_{378}O_{379}S_{12}$	30,454.99	30,434.93	Δ 128.27	-20.81
	24 mer	$C_{1383}H_{2168}N_{380}O_{380}S_{12}$	30,583.16	30,563.20	Δ 127.59	-19.96
	25 mer	$C_{1389}H_{2180}N_{382}O_{381}S_{12}$	30,711.33	30,690.79	Δ 129.56	-20.54
	26 mer	$C_{1395}H_{2192}N_{384}O_{382}S_{12}$	30,839.51	30,820.35	Δ 127.20	-19.16
	27 mer	$C_{1401}H_{2204}N_{386}O_{383}S_{12}$	30,967.68	30,947.55		-20.13

[a] The average molecular masses are shown.

[b] The molecular mass of the L-αLys monomer unit is calculated to be 128.095 Da.

[c] Unexpected MS discrepancies (about -20 Da) between the calculated and observed MSs, suggesting the post-translation modification of the recombinant mAG during the overexpression in *E. coli.*

Supplementary Table 14

The HPLC-HR-ESI-MS data of R8-mAG (**13**) chemically synthesized

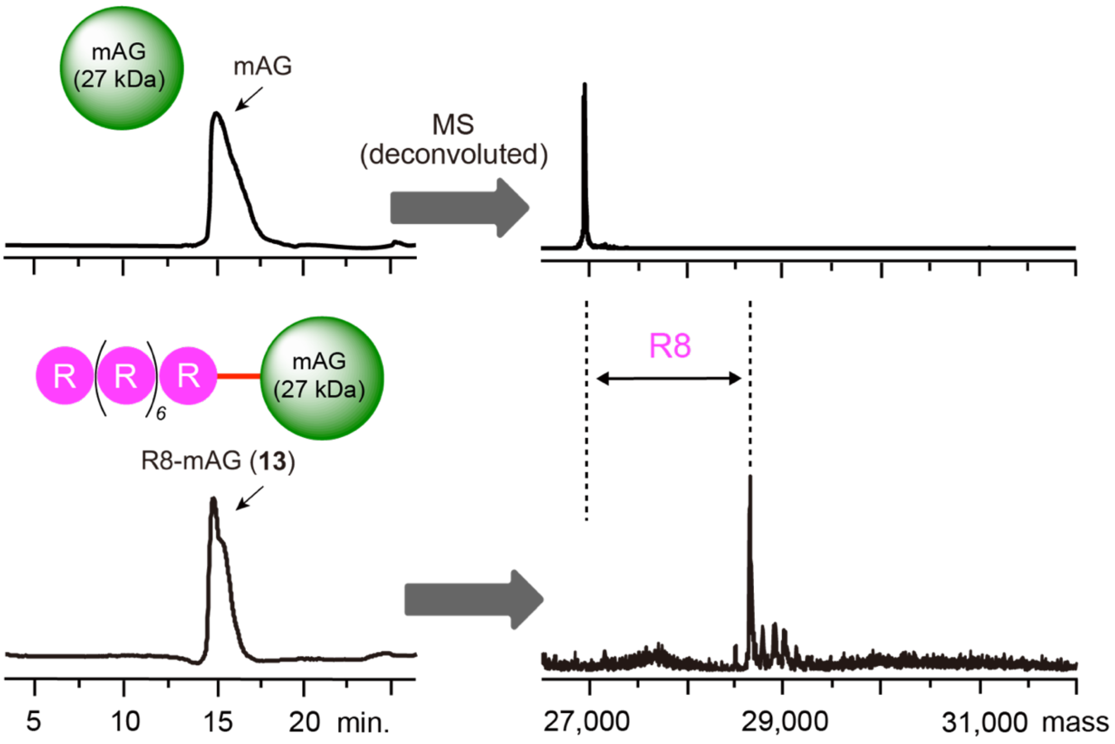

The total ion current (TIC) chromatograms (Right) and the deconvoluted MS spectra (Right) are shown.

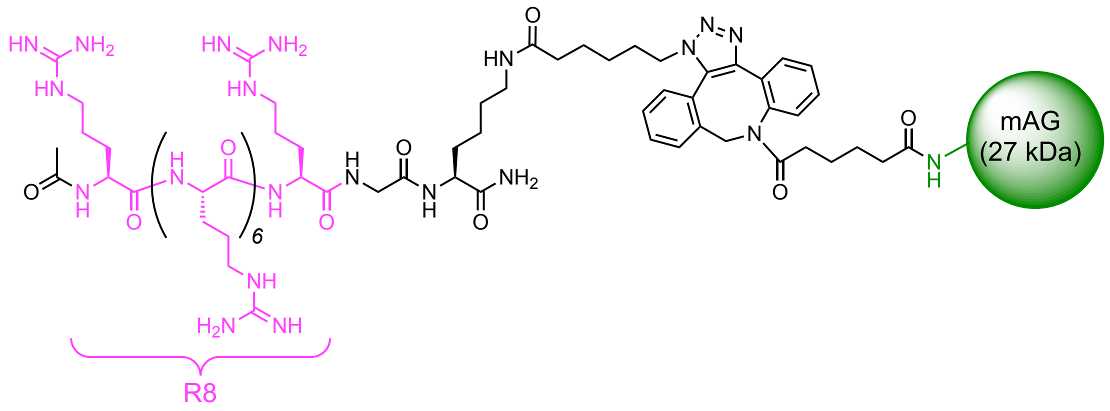

compound (**ID**)	peptide -chain length	molecular formula	calculated mass [a]	observed mass (deconvoluted) [a]	mass discrepancy [b] (Da)
mAG	N/A	$C_{1210}H_{1846}N_{328}O_{350}S_{12}$	26,972.42	26,951.97	-20.45
**13**	8 mer	$C_{1295}H_{1988}N_{368}O_{364}S_{12}$	28,920.21	28,899.50	-20.71

[a] The average molecular masses are shown.
[b] Unexpected MS discrepancies (about -20 Da) between the calculated and observed MSs, suggesting the post-translation modification of the recombinant mAG during the overexpression in *E. coli.*

Supplementary Table 15

The mass data of ε-PαL-mKO (**14**) chemically synthesized

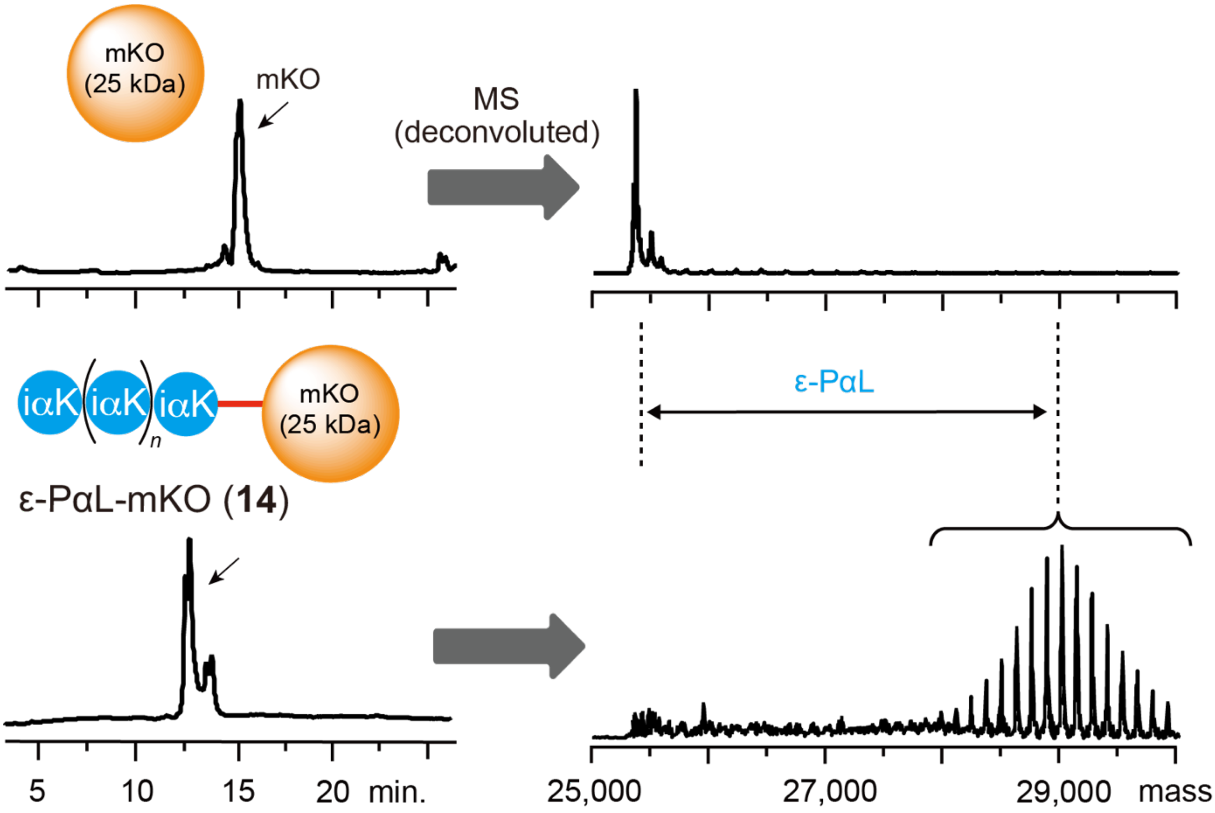

The total ion current (TIC) chromatograms (Right) and the deconvoluted MS spectra (Right) are shown.

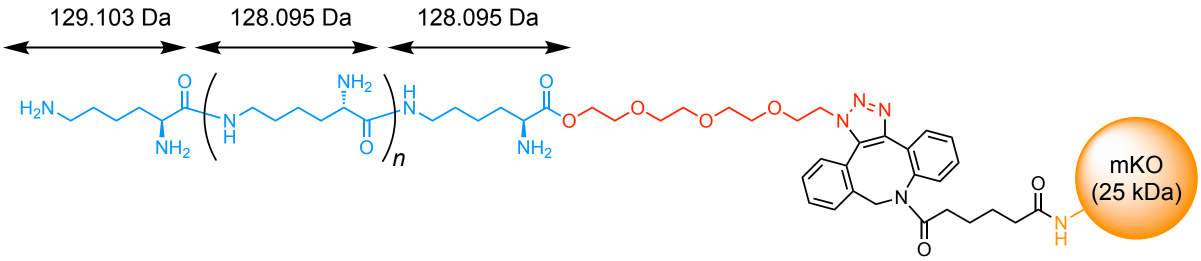

(Continued on the next page)

compound (**ID**)	peptide -chain length	molecular formula	calculated mass [a]	observed mass (deconvoluted) [a]	monomer-unit difference [b] (Da)	mass discrepancy [c] (Da)
mKO	N/A	$C_{1134}H_{1717}N_{311}O_{333}S_{11}$	25,387.41	25,365.84	N/A	-21.57
**14**	17 mer	$C_{1265}H_{1955}N_{349}O_{356}S_{11}$	28,100.94	28,080.40		-20.54
	18 mer	$C_{1271}H_{1967}N_{351}O_{357}S_{11}$	28,229.12	28,207.81	Δ 127.41	-21.31
	19 mer	$C_{1277}H_{1979}N_{353}O_{358}S_{11}$	28,357.29	28,336.17	Δ 128.36	-21.12
	20 mer	$C_{1283}H_{1991}N_{355}O_{359}S_{11}$	28,485.46	28,463.24	Δ 127.07	-22.22
	21 mer	$C_{1289}H_{2003}N_{357}O_{360}S_{11}$	28,613.63	28,593.23	Δ 129.99	-20.40
	22 mer	$C_{1295}H_{2015}N_{359}O_{361}S_{11}$	28,741.81	28,721.42	Δ 128.19	-20.39
	23 mer	$C_{1301}H_{2027}N_{361}O_{362}S_{11}$	28,869.98	28,849.82	Δ 128.40	-20.16
	24 mer	$C_{1307}H_{2039}N_{363}O_{363}S_{11}$	28,998.15	28,977.85	Δ 128.03	-20.30
	25 mer	$C_{1313}H_{2051}N_{365}O_{364}S_{11}$	29,126.32	29,106.28	Δ 128.43	-20.04
	26 mer	$C_{1319}H_{2063}N_{367}O_{365}S_{11}$	29,254.50	29,233.81	Δ 127.53	-20.69
	27 mer	$C_{1325}H_{2075}N_{369}O_{366}S_{11}$	29382.67	29,362.73	Δ 128.92	-19.94

[a] The average molecular masses are shown.
[b] The molecular mass of the L-αLys monomer unit is calculated to be 128.095 Da.
[c] Unexpected MS discrepancies (about -20 Da) between the calculated and observed MSs, suggesting the post-translation modification of the recombinant mKO during the overexpression in *E. coli.*

Supplementary Table 16

The HPLC-HR-ESI-MS data of R8-mKO (**15**) chemically synthesized

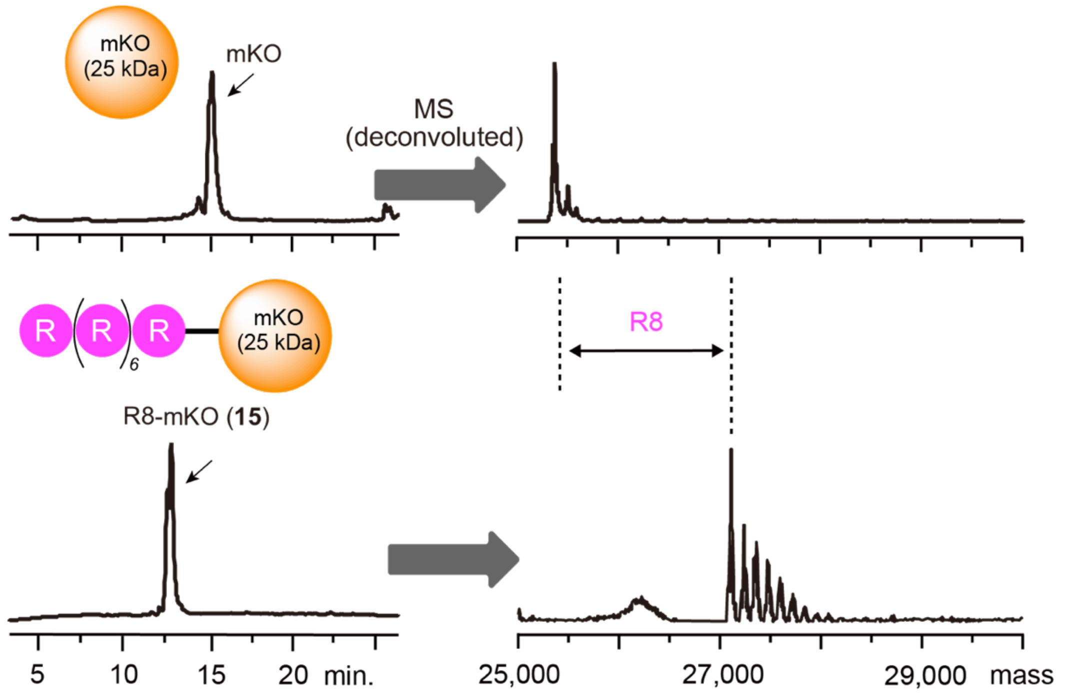

The total ion current (TIC) chromatograms (Right) and the deconvoluted MS spectra (Right) are shown.

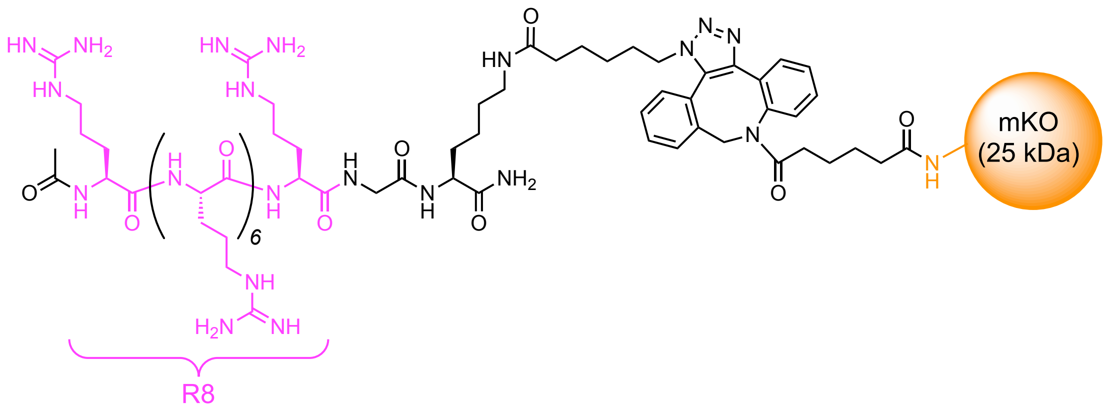

compound (**ID**)	peptide -chain length	molecular formula	calculated mass [a]	observed mass (deconvoluted) [a]	mass discrepancy [b] (Da)
mKO	N/A	$C_{1134}H_{1717}N_{311}O_{333}S_{11}$	25,387.41	25,365.84	-21.57
**15**	8 mer	$C_{1219}H_{1859}N_{351}O_{347}S_{11}$	27,335.71	27,314.75	-20.96

[a] The average molecular masses are shown.
[b] Unexpected MS discrepancies (about -20 Da) between the calculated and observed MSs, suggesting the post-translation modification of the recombinant mKO during the overexpression in *E. coli.*

Supplementary Table 17

The HPLC-HR-ESI-MS data of ε-PαL-Cre (**16**) chemically synthesized

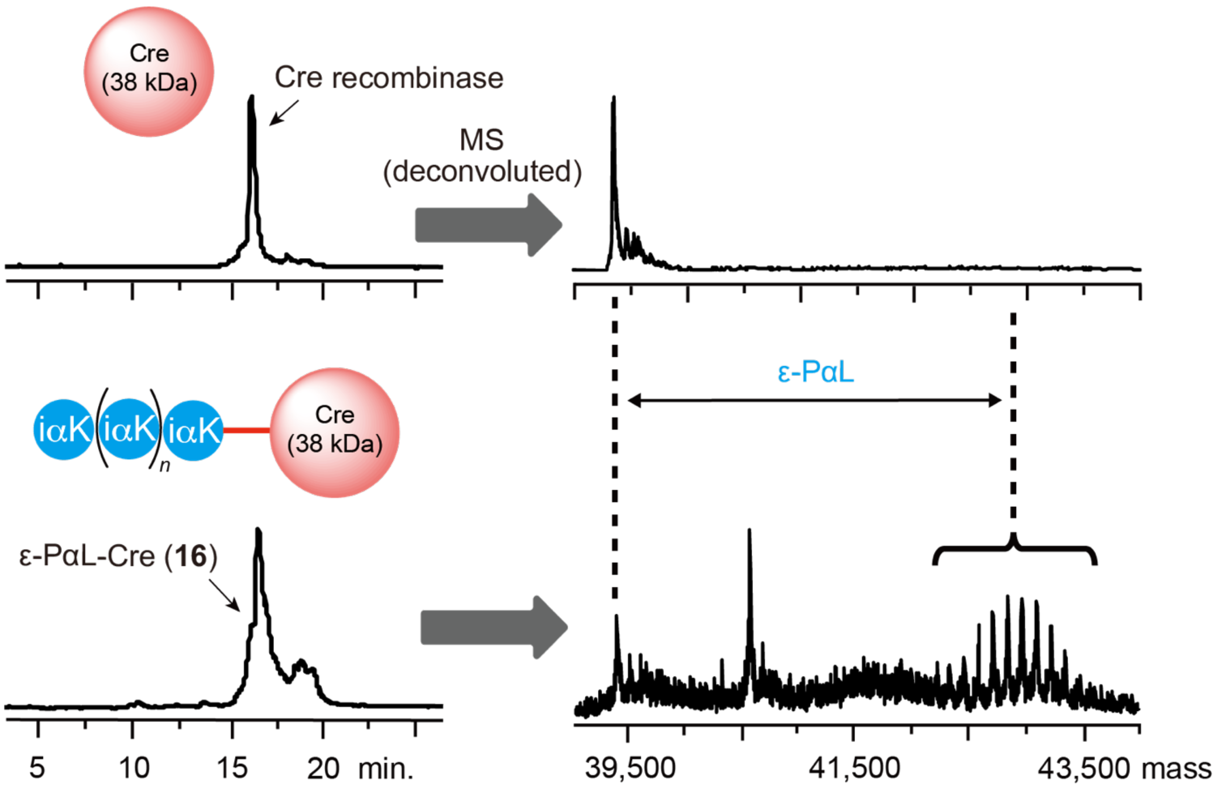

The total ion current (TIC) chromatograms (Right) and the deconvoluted MS spectra (Right) are shown.

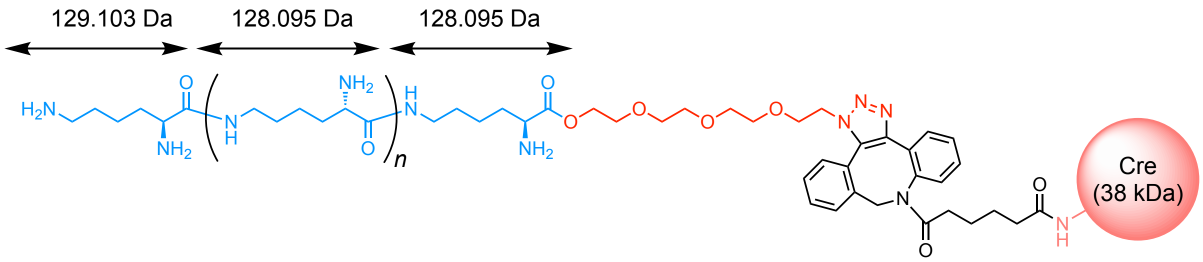

(Continued on the next page)

compound (**ID**)	peptide -chain length	molecular formula	calculated mass [a]	observed mass (deconvoluted) [a]	monomer-unit difference [b] (Da)	mass discrepancy [c] (Da)
Cre	N/A	$C_{1710}H_{2749}N_{529}O_{502}S_{15}$	39,231.41	39,361.32	N/A	+129.91
**16**	19 mer	$C_{1853}H_{3011}N_{571}O_{527}S_{15}$	42,201.29	42,328.82		+127.53
	20 mer	$C_{1859}H_{3023}N_{573}O_{528}S_{15}$	42,329.46	42,458.59	Δ 129.77	+129.13
	21 mer	$C_{1865}H_{3035}N_{575}O_{529}S_{15}$	42,457.63	42,588.40	Δ 129.81	+130.77
	22 mer	$C_{1871}H_{3047}N_{577}O_{530}S_{15}$	42,585.81	42,714.52	Δ 126.12	+128.71
	23 mer	$C_{1877}H_{3059}N_{579}O_{531}S_{15}$	42,713.98	42,844.61	Δ 130.09	+130.63
	24 mer	$C_{1883}H_{3071}N_{581}O_{532}S_{15}$	42,842.15	42,972.99	Δ 128.38	+130.84
	25 mer	$C_{1889}H_{3083}N_{583}O_{533}S_{15}$	42,970.32	43,101.12	Δ 128.13	+130.80
	26 mer	$C_{1895}H_{3095}N_{585}O_{534}S_{15}$	43,098.50	43,229.92	Δ 128.80	+131.42
	27 mer	$C_{1901}H_{3107}N_{587}O_{535}S_{15}$	43,226.67	43,355.58	Δ 125.66	+128.91

[a] The average molecular masses are shown.

[b] The molecular mass of the L-αLys monomer unit is calculated to be 128.095 Da.

[c] Unexpected MS discrepancies (about +129 Da) between the calculated and observed MSs, suggesting the post-translation modification of the recombinant Cre recombinase during the overexpression in *E. coli.*

Supplementary Table 18

The HPLC-HR-ESI-MS data of ε-PαL-mAb^{FAM} (**17**) chemically synthesized

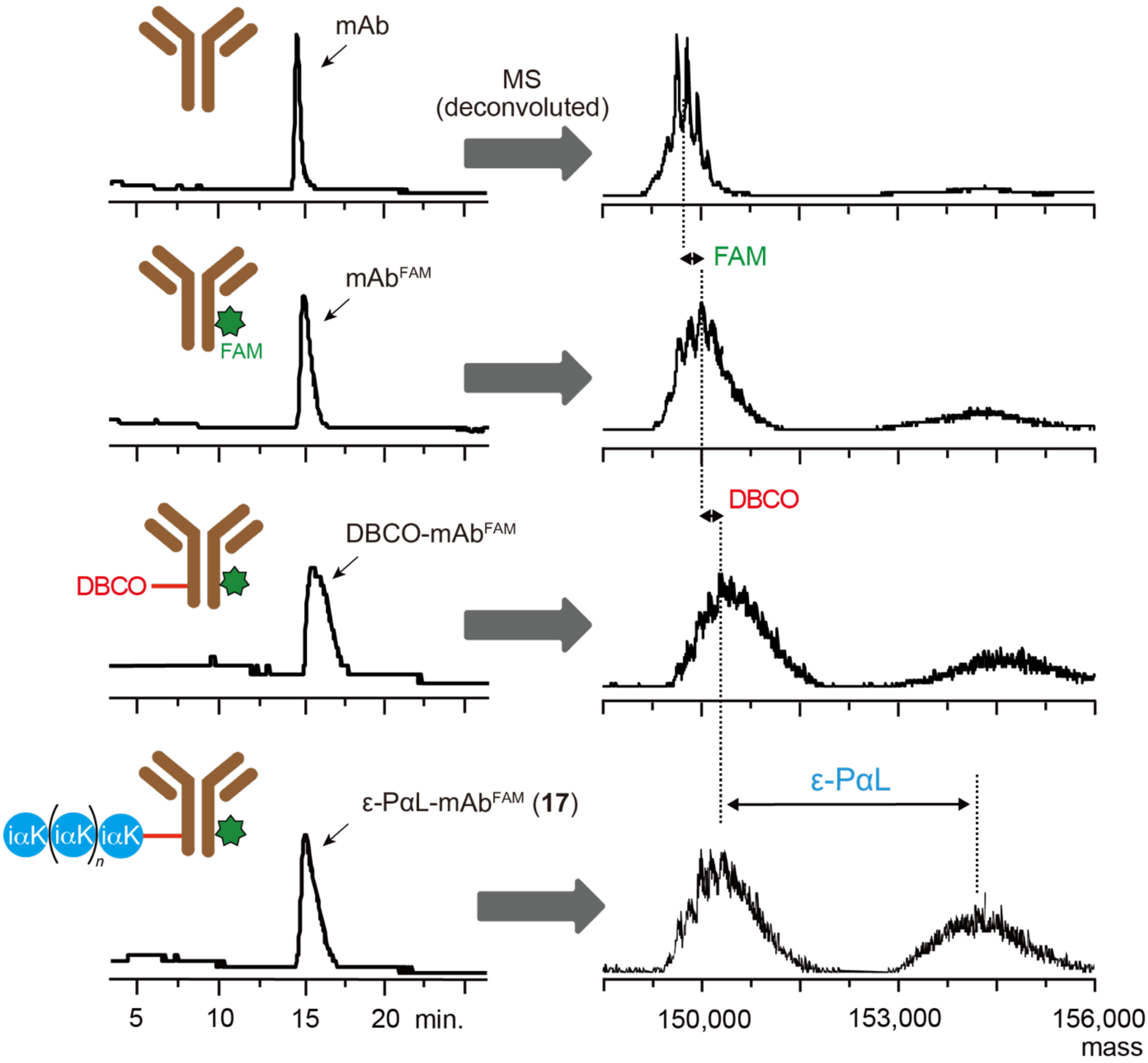

The total ion current (TIC) chromatograms (Right) and the deconvoluted MS spectra (Right) are shown.

(Continued on the next page)

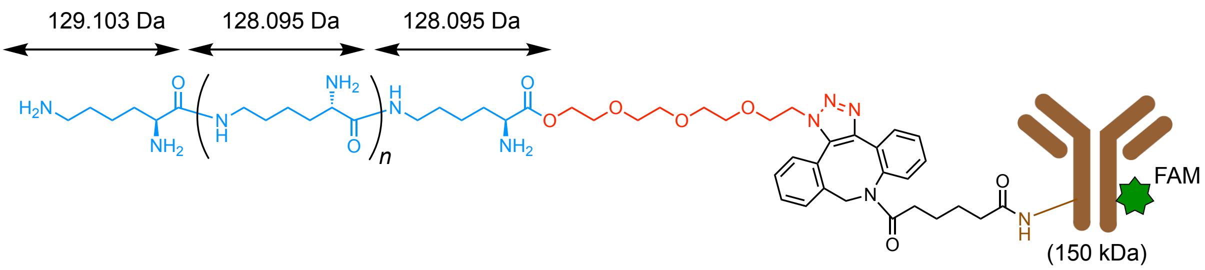

compound (**ID**)	ε-PαL isopeptide chain length	molecular weight (kDa)
mAb	N/A	149.6
mAb^{FAM}	N/A	150.0
DBCO-mAb^{FAM}	N/A	150.3
**17**	19 - 27 mer	153.0 - 155.5

Supplementary Table 19

Cytotoxicities of ε-PαL (**1**)

Cell line	IC_{50} (μM)	
	actinomycin D	ε-PαL (**1**)
K562	3.00×10^{-3}	>100
HeLa	4.92×10^{-4}	>100
HepG2	6.81×10^{-4}	>100
HEK293T	6.38×10^{-6}	>100

Cytotoxicities against mammalian cells were determined by a colorimetric assay, using 2-(2-methoxy-4-nitrophenyl)-3-(4-nitrophenyl)-5-(2,4-disulfophenyl)-2*H*-tetrazolium monosodium salt (WST-8). Cell lines were grown at 37°C in a humidified atmosphere with 5% CO_2. The cell lines and corresponding media were as follows: HeLa, Dulbecco's modified Eagles Medium (DMEM) with 10% fetal bovine serum (FBS); HEK293T, DMEM with 10% FBS; HepG2, DMEM with 10% FBS; and K562, RPMI1640 with 10% FBS. All media contain 100 U/ml penicillin and 100 μg/ml streptomycin. The 384-well plates were seeded with aliquots of 20 μl medium containing 1×10^3 cells per well, and were incubated overnight before being treated with ε-PαL (**1**) or actinomycin D (positive control) at various concentrations for 48 h. Plates were incubated for 1 h at 37 °C after the addition of 2 μl of WST-8 reagent solution (Cell Counting Kit; Dojindo) per well. The absorption of the formed formazan dye was measured at 450 nm.

Supplementary Table 20

Elucidation of the chemical structure of L-βLys-PEG-azide (**18**) by NMR

^{13}C (150 MHz) and ^{1}H (600 MHz) NMR data[a] for L-βLys-PEG-azide (**18**)

No.	δ_C	δ_H (multiplicity, J = Hz)
1	53.0	3.50 (t, J = 5.2)
2	72.2	3.71 (m)
3	72.2	3.71 (m)
4	72.2	3.71 (m)
5	72.2	3.71 (m)
6	72.2	3.71 (m)
7	71.5	3.64 (m)
8	41.7	3.41 (t, J = 6.1)
9	174.5	
10	39.4	2.48 (dd, J = 16.8, 7.1)
		2.58 (dd, J = 16.4, 5.4)
11	51.2	3.64 (m)
12	31.8	1.78 (m)
13	25.7	1.78 (m)
14	41.6	3.03 (t, J = 8.3)

[a] NMR spectra were obtained with the JNM-ECP600 in D_2O including 0.1% TMSP(trimethylsilyl propanoic acid). Water signal was used as an internal standard (δ_H 4.79 ppm). TMSP signal was used as an internal standard (δ_C 0.0 ppm).

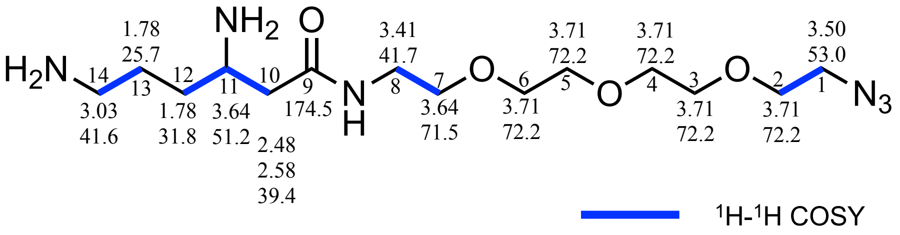

Supplementary Table 21

The mass data of ε-OβL-PEG-azide (**19**) chemoenzymatically synthesized by rORF19.

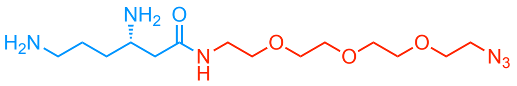

L-βLys-PEG-azide (**18**)

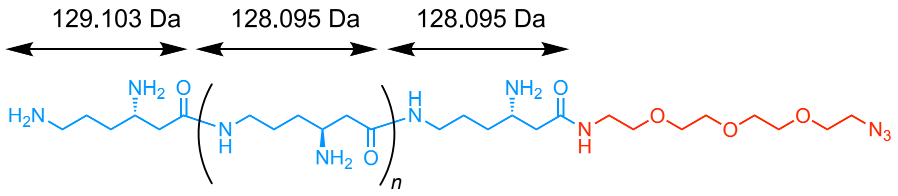

ε-OβL-PEG-azide (**19**)

compound (**ID**)	peptide -chain length	molecular formula	calculated m/z value [a]	observed m/z value [a]	monomer-unit difference [b] (Da)
**18**	N/A	$C_{14}H_{30}N_6O_4$	347.240 $[M+H]^+$	347.242 $[M+H]^+$	
					Δ 128.095
**19**	2 mer	$C_{20}H_{42}N_8O_5$	475.335 $[M+H]^+$	475.337 $[M+H]^+$	
					Δ 128.096
	3 mer	$C_{26}H_{54}N_{10}O_6$	603.430 $[M+H]^+$	603.433 $[M+H]^+$	
					Δ 128.095
	4 mer	$C_{32}H_{66}N_{12}O_7$	731.525 $[M+H]^+$	731.528 $[M+H]^+$	

[a] The values are shown for the monoisotopic masses.
[b] The molecular mass of the L-αLys monomer unit is calculated to be 128.095 Da.

Supplementary Table 22

Elucidation of the chemical structure of L-βhLys-PEG-azide (**20**) by NMR.

^{13}C (150 MHz) and ^{1}H (600 MHz) NMR data[a] for L-βhLys-PEG-azide (**20**)

No.	δ_C	δ_H (multiplicity, J = Hz)
1	53.0	3.50 (t, J = 4.9)
2	72.3	3.73 (m)
3	72.3	3.73 (m)
4	72.3	3.73 (m)
5	72.3	3.73 (m)
6	72.3	3.73 (m)
7	71.5	3.64 (m)
8	41.8	3.41 (t, J = 5.4)
9	174.7	
10	39.6	2.61 (dd, J = 16.0, 7.7)
		2.70 (dd, J = 16.0, 5.1)
11	51.5	3.64 (m)
12	34.3	1.72 (m)
13	24.5	1.48 (m)
14	29.2	1.72 (m)
15	41.9	3.01 (t, J = 7.2)

[a] NMR spectra were obtained with the JNM-ECP600 in D_2O including 0.1% TMSP(trimethylsilyl propanoic acid). Water signal was used as an internal standard (δ_H 4.79 ppm). TMSP signal was used as an internal standard (δ_C 0.0 ppm).

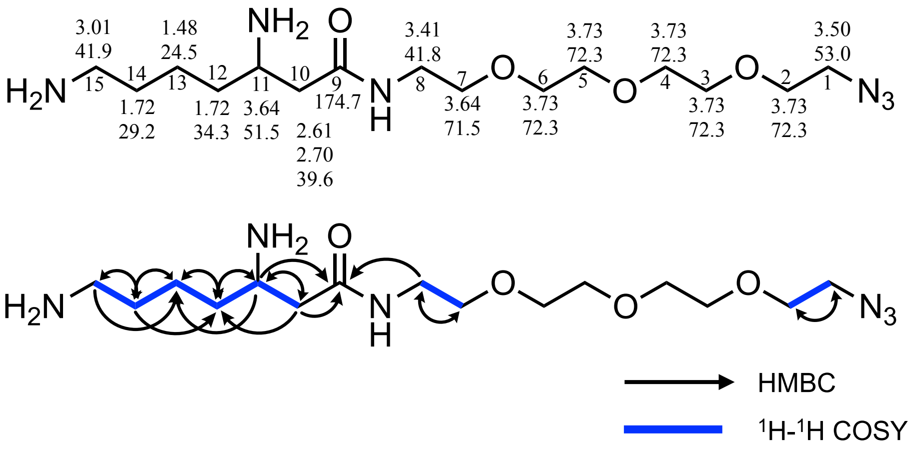

Supplementary Table 23

The mass data of ε-OβL^{m}-PEG-azide (**21**) chemoenzymatically synthesized by rORF19.

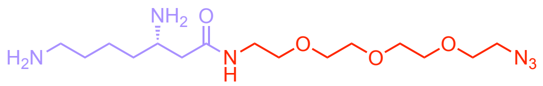

L-βhLys-PEG-azide (**20**)

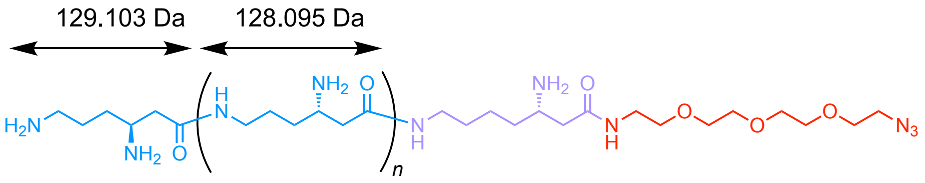

ε-OβL^{m}-PEG-azide (**21**)

compound (**ID**)	peptide -chain length	molecular formula	calculated m/z value [a]	observed m/z value [a]	monomer-unit difference [b] (Da)
**20**	1 mer	$C_{15}H_{32}N_6O_4$	361.256 $[M+H]^+$	361.254 $[M+H]^+$	
**21**	2 mer	$C_{21}H_{44}N_8O_5$	489.351 $[M+H]^+$	489.353 $[M+H]^+$	Δ 128.099
	3 mer	$C_{27}H_{56}N_{10}O_6$	617.446 $[M+H]^+$	617.448 $[M+H]^+$	Δ 128.095
	4 mer	$C_{33}H_{68}N_{12}O_7$	745.541 $[M+H]^+$	745.543 $[M+H]^+$	Δ 128.095

[a] The values are shown for the monoisotopic masses.
[b] The molecular mass of the L-αLys monomer unit is calculated to be 128.095 Da.

Supplementary Table 24

Elucidation of the chemical structure of L-αLys-PEG-azide (**22**) by NMR.

^{13}C (150 MHz) and ^{1}H (600 MHz) NMR data[a] for L-αLys-PEG-azide (**22**)

No.	δ_C	δ_H (multiplicity, J = Hz)
1	53.1	3.50 (t, J = 4.8)
2	72.3	3.71 (m)
3	72.3	3.71 (m)
4	72.3	3.71 (m)
5	72.3	3.71 (m)
6	72.3	3.71 (m)
7	71.5	3.65 (t, J = 5.3)
8	42.0	3.46 (t, J = 5.3)
9	172.6	
10	56.1	3.97 (t, J = 6.6)
11	33.3	1.91 (m)
12	24.2	1.45 (m)
13	29.3	1.71 (m)
14	41.8	3.00 (t, J = 7.6)

[a] NMR spectra were obtained with the JNM-ECP600 in D_2O including 0.1% TMSP(trimethylsilyl propanoic acid). Water signal was used as an internal standard (δ_H 4.79 ppm). TMSP signal was used as an internal standard (δ_C 0.0 ppm).

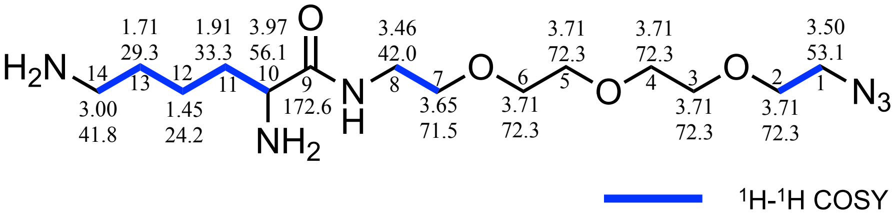

Supplementary Table 25

The HPLC-HR-ESI-MS data of ε-OβL^{m}-PEG-azide (**21**) enzymatically synthesized (1st round reaction and 2nd round reaction).

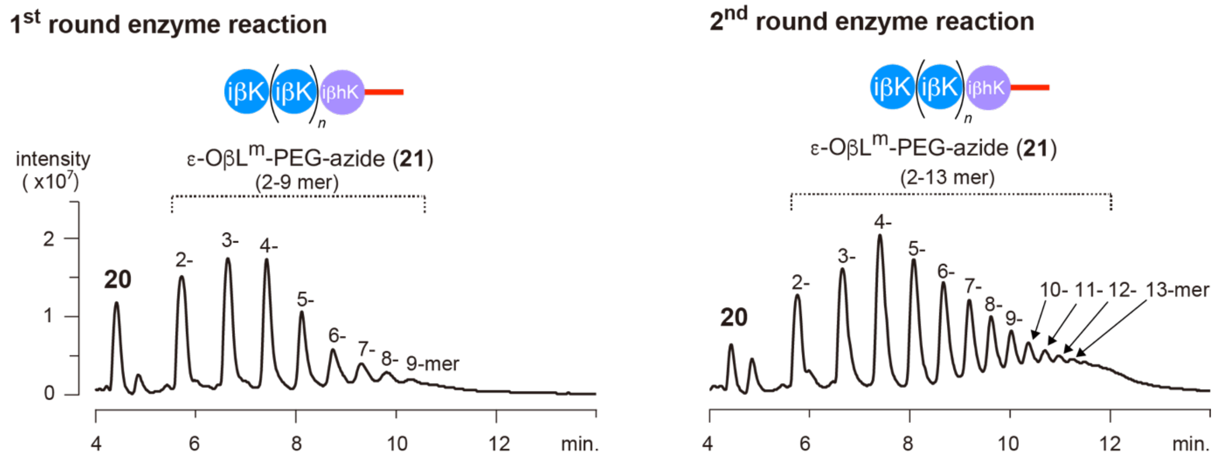

The 1st round reaction (left) and 2nd round reaction (right) mixtures were analyzed by HPLC-HR-ESI-MS. The total ion current (TIC) chromatograms are shown.

(Continued on the next page)

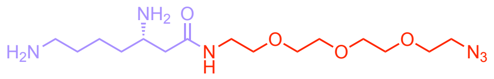

L-βhLys-PEG-azide (**20**)

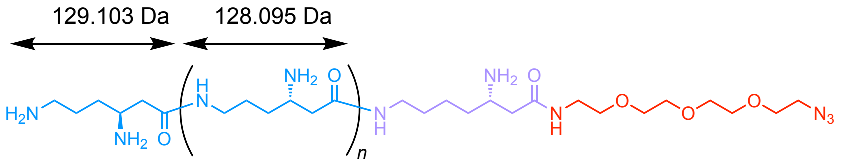

ε-OβL^{m}-PEG-azide (**21**)

compound (**ID**)	peptide -chain length	molecular formula	calculated *m/z* value [a]	observed *m/z* value [a]	observed mass (deconvoluted) [a]	monomer-unit difference [b] (Da)
**1st round enzyme reaction**						
**20**	1 mer	$C_{15}H_{32}N_6O_4$	361.249 $[M+H]^+$	361.253 $[M+H]^+$	N/A	N/A
**21**	2 mer	$C_{21}H_{44}N_8O_5$	489.351 $[M+H]^+$	489.349 $[M+H]^+$	N/A	N/A
	3 mer	$C_{27}H_{56}N_{10}O_6$	617.446 $[M+H]^+$	617.443 $[M+H]^+$	N/A	N/A
	4 mer	$C_{33}H_{68}N_{12}O_7$	745.541 $[M+H]^+$	745.537 $[M+H]^+$	N/A	N/A
	5 mer	$C_{39}H_{80}N_{14}O_8$	873.636 $[M+H]^+$	873.631 $[M+H]^+$	N/A	N/A
	6 mer	$C_{45}H_{92}N_{16}O_9$	501.369 $[M+2H]^{2+}$	501.367 $[M+2H]^{2+}$	N/A	N/A
	7 mer	$C_{51}H_{104}N_{18}O_{10}$	565.416 $[M+2H]^{2+}$	565.417 $[M+2H]^{2+}$	N/A	N/A
	8 mer	$C_{57}H_{116}N_{20}O_{11}$	629.464 $[M+2H]^{2+}$	629.464 $[M+2H]^{2+}$	N/A	N/A
	9 mer	$C_{63}H_{128}N_{22}O_{12}$	693.511 $[M+2H]^{2+}$	693.513 $[M+2H]^{2+}$	N/A	N/A
**2nd round enzyme reaction**						
**20**	1 mer	$C_{15}H_{32}N_6O_4$	361.249 $[M+H]^+$	361.254 $[M+H]^+$	360.249	
**21**	2 mer	$C_{21}H_{44}N_8O_5$	489.351 $[M+H]^+$	489.349 $[M+H]^+$	488.347	Δ 128.098
	3 mer	$C_{27}H_{56}N_{10}O_6$	617.446 $[M+H]^+$	617.444 $[M+H]^+$	616.439	Δ 128.092
	4 mer	$C_{33}H_{68}N_{12}O_7$	745.541 $[M+H]^+$	745.538 $[M+H]^+$	744.534	Δ 128.098
	5 mer	$C_{39}H_{80}N_{14}O_8$	873.636 $[M+H]^+$	873.633 $[M+H]^+$	872.629	Δ 128.095
	6 mer	$C_{45}H_{92}N_{16}O_9$	501.369 $[M+2H]^{2+}$	501.368 $[M+2H]^{2+}$	1,000.723	Δ 128.094
	7 mer	$C_{51}H_{104}N_{18}O_{10}$	565.416 $[M+2H]^{2+}$	565.415 $[M+2H]^{2+}$	1,128.819	Δ 128.096
	8 mer	$C_{57}H_{116}N_{20}O_{11}$	629.464 $[M+2H]^{2+}$	629.463 $[M+2H]^{2+}$	1,256.916	Δ 128.097
	9 mer	$C_{63}H_{128}N_{22}O_{12}$	693.511 $[M+2H]^{2+}$	693.510 $[M+2H]^{2+}$	1,385.009	Δ 128.093
	10 mer	$C_{69}H_{140}N_{24}O_{13}$	505.375 $[M+3H]^{3+}$	505.374 $[M+3H]^{3+}$	1,513.095	Δ 128.086
	11 mer	$C_{75}H_{152}N_{26}O_{14}$	548.073 $[M+3H]^{3+}$	548.072 $[M+3H]^{3+}$	1,641.194	Δ 128.099
	12 mer	$C_{81}H_{164}N_{28}O_{15}$	590.772 $[M+3H]^{3+}$	590.772 $[M+2H]^{2+}$	1,769.284	Δ 128.090
	13 mer	$C_{87}H_{176}N_{30}O_{16}$	633.470 $[M+3H]^{3+}$	633.471 $[M+3H]^{3+}$	1,897.386	Δ 128.102

[a] The values of *m/z* and molecular mass are shown for the monoisotopic ion peaks.

[b] The molecular mass of the L-βLys monomer unit is calculated to be 128.095 Da.

Supplementary Table 26

The HPLC-HR-ESI-MS data of the ε-OβL^{m}-FAM (**24**) compounds with different isopeptide chain lengths (2–13 mer), which were chemically synthesized.

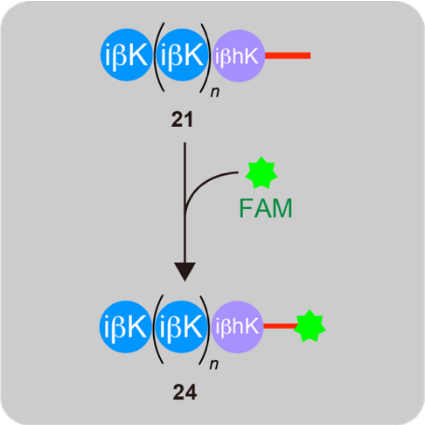

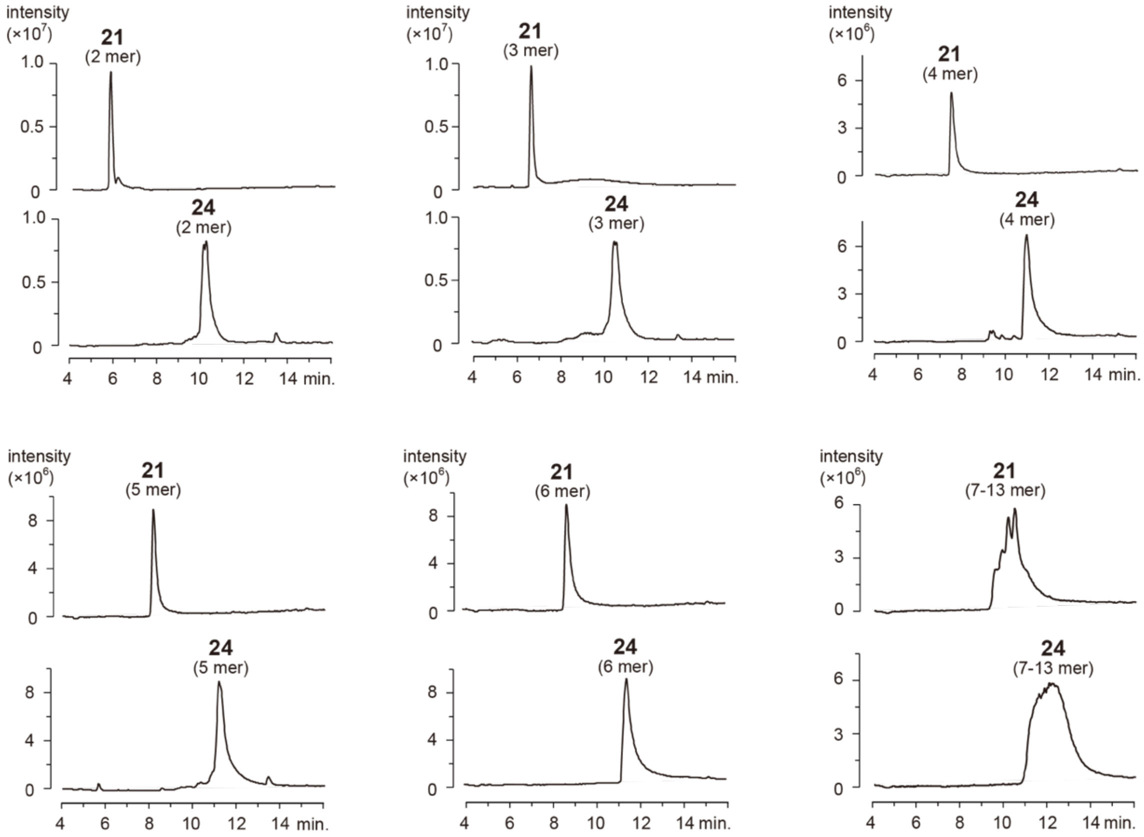

The chemically synthesized ε-OβL^{m}-FAM (**24**) compounds with different isopeptide chain lengths were analyzed by HPLC-HR-ESI-MS. The total ion current (TIC) chromatograms are shown.

(Continued on the next page)

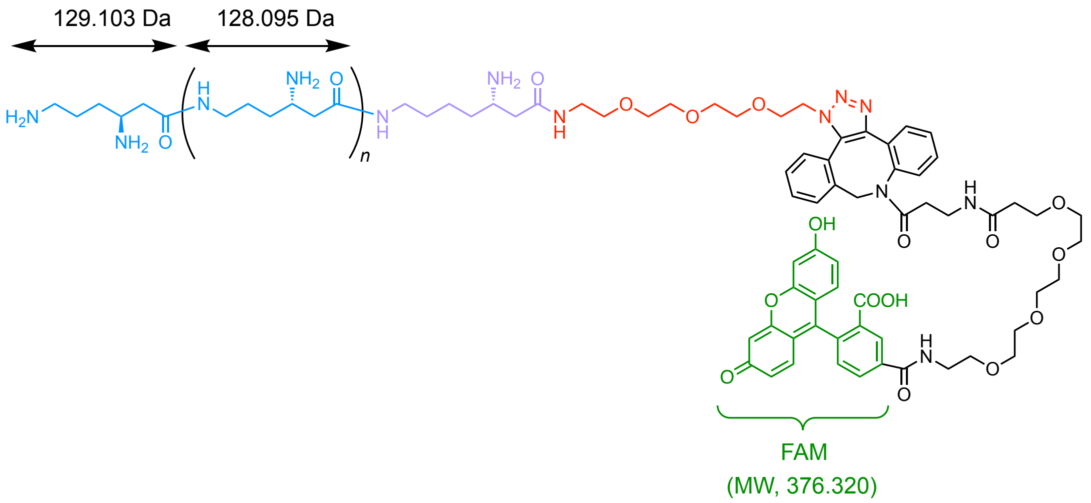

compound (**ID**)	peptide -chain length	molecular formula	calculated *m/z* value [a]	observed *m/z* value [a]	observed mass (deconvoluted) [a]	monomer-unit difference [b] (Da)
**24**	2 mer	$C_{71}H_{91}N_{11}O_{17}$	685.837 $[M+2H]^{2+}$	685.837 $[M+2H]^{2+}$	1,369.659 [M]	
						Δ 128.099
	3 mer	$C_{77}H_{103}N_{13}O_{18}$	749.885 $[M+2H]^{2+}$	749.885 $[M+2H]^{2+}$	1,497.758 [M]	
						Δ 128.094
	4 mer	$C_{83}H_{115}N_{15}O_{19}$	542.957 $[M+3H]^{3+}$	542.958 $[M+3H]^{3+}$	1,625.852 [M]	
						Δ 128.095
	5 mer	$C_{89}H_{127}N_{17}O_{20}$	585.655 $[M+3H]^{3+}$	585.656 $[M+3H]^{3+}$	1,753.947 [M]	
						Δ 128.094
	6 mer	$C_{95}H_{139}N_{19}O_{21}$	628.354 $[M+3H]^{3+}$	628.354 $[M+3H]^{3+}$	1,882.041 [M]	
	7-13 mer	$C_{101}H_{151}N_{21}O_{22}$	671.052 $[M+3H]^{3+}$	671.052 $[M+3H]^{3+}$	2,010.133 [M]	
						Δ 128.098
		$C_{107}H_{163}N_{23}O_{23}$	713.750 $[M+3H]^{3+}$	713.751 $[M+3H]^{3+}$	2,138.231 [M]	
						Δ 128.090
		$C_{113}H_{175}N_{25}O_{24}$	756.449 $[M+3H]^{3+}$	756.449 $[M+3H]^{3+}$	2,266.321 [M]	
						Δ 128.093
		$C_{119}H_{187}N_{27}O_{25}$	799.147 $[M+3H]^{3+}$	799.147 $[M+3H]^{3+}$	2,394.414 [M]	
						Δ 128.094
		$C_{125}H_{199}N_{29}O_{26}$	841.845 $[M+3H]^{3+}$	841.845 $[M+3H]^{3+}$	2,522.508 [M]	
						Δ 128.084
		$C_{131}H_{211}N_{31}O_{27}$	884.544 $[M+3H]^{3+}$	884.544 $[M+3H]^{3+}$	2,650.592 [M]	
		$C_{137}H_{223}N_{33}O_{28}$	927,242 $[M+3H]^{3+}$	(weak signal)		

[a] The values of *m/z* and molecular mass are shown for the monoisotopic ion peaks.

[b] The molecular mass of the L-βLys monomer unit is calculated to be 128.095 Da.

Supplementary Table 27

The HPLC-HR-ESI-MS data of ε-OβL^{m}-FAM (**24**) consisting of 4–13 mer, which was chemically synthesized.

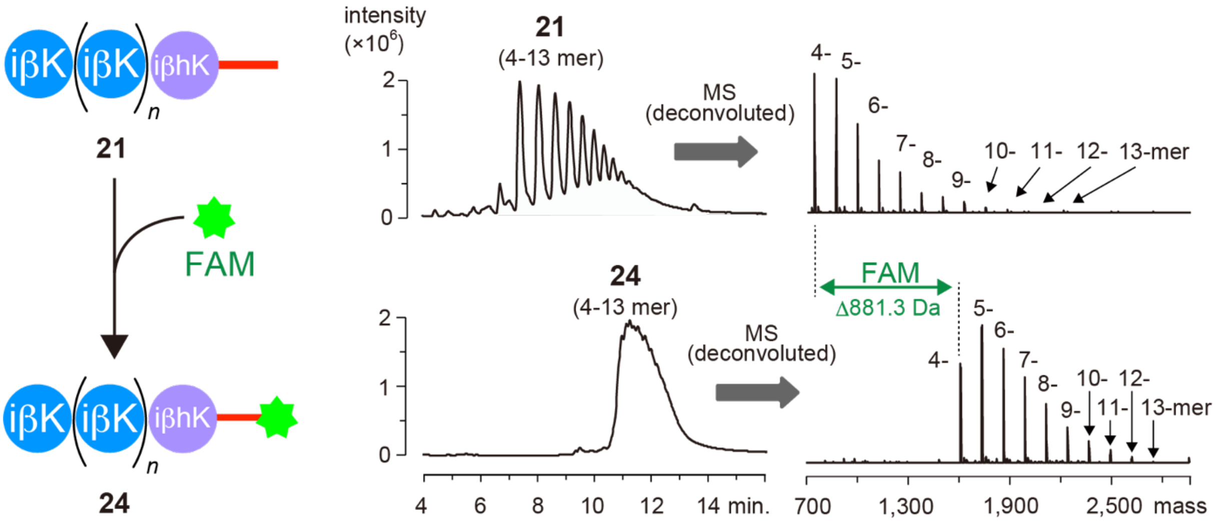

The chemically synthesized ε-OβL^{m}-FAM (**24**) consisting of 4-13 mer was analyzed by HPLC-HR-ESI-MS. The total ion current (TIC) chromatograms are shown.

(Continued on the next page)

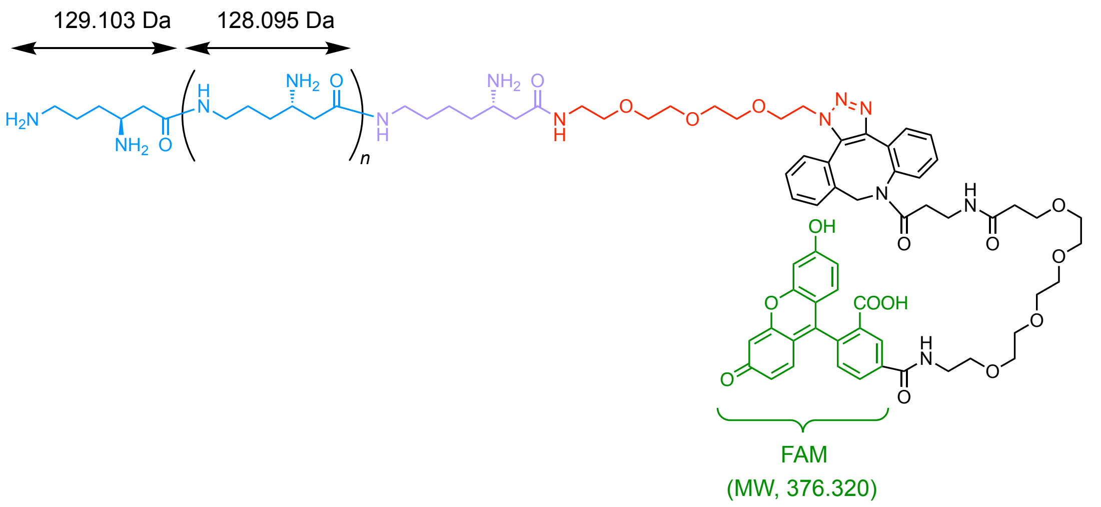

compound (**ID**)	peptide -chain length	molecular formula	calculated *m/z* value [a]	observed *m/z* value [a]	observed mass (deconvoluted) [a]	monomer-unit difference [b] (Da)
**24**	4-13 mer	$C_{83}H_{115}N_{15}O_{19}$	542.957 $[M+3H]^{3+}$	542.958 $[M+3H]^{3+}$	1,625.852 [M]	Δ 128.096
		$C_{89}H_{127}N_{17}O_{20}$	585.655 $[M+3H]^{3+}$	585.656 $[M+3H]^{3+}$	1,753.948 [M]	Δ 128.094
		$C_{95}H_{139}N_{19}O_{21}$	628.354 $[M+3H]^{3+}$	628.355 $[M+3H]^{3+}$	1,882.042 [M]	Δ 128.096
		$C_{101}H_{151}N_{21}O_{22}$	671.052 $[M+3H]^{3+}$	671.053 $[M+3H]^{3+}$	2,010.138 [M]	Δ 128.094
		$C_{107}H_{163}N_{23}O_{23}$	713.750 $[M+3H]^{3+}$	713.751 $[M+3H]^{3+}$	2,138.232 [M]	Δ 128.092
		$C_{113}H_{175}N_{25}O_{24}$	756.449 $[M+3H]^{3+}$	756.450 $[M+3H]^{3+}$	2,266.324 [M]	Δ 128.096
		$C_{119}H_{187}N_{27}O_{25}$	799.147 $[M+3H]^{3+}$	799.148 $[M+3H]^{3+}$	2,394.420 [M]	Δ 128.098
		$C_{125}H_{199}N_{29}O_{26}$	841.845 $[M+3H]^{3+}$	841.846 $[M+3H]^{3+}$	2,522.518 [M]	Δ 128.091
		$C_{131}H_{211}N_{31}O_{27}$	884.544 $[M+3H]^{3+}$	884.545 $[M+3H]^{3+}$	2,650.609 [M]	
		$C_{137}H_{223}N_{33}O_{28}$	927,242 $[M+3H]^{3+}$	(weak signal)		

[a] The values of *m/z* and molecular mass are shown for the monoisotopic ion peaks.

[b] The molecular mass of the L-βLys monomer unit is calculated to be 128.095 Da.

Supplementary Table 28

The HPLC-HR-ESI-MS data of ε-OβL^{m}-mAG (**25**) consisting of 4–13 mer, which was chemically synthesized.

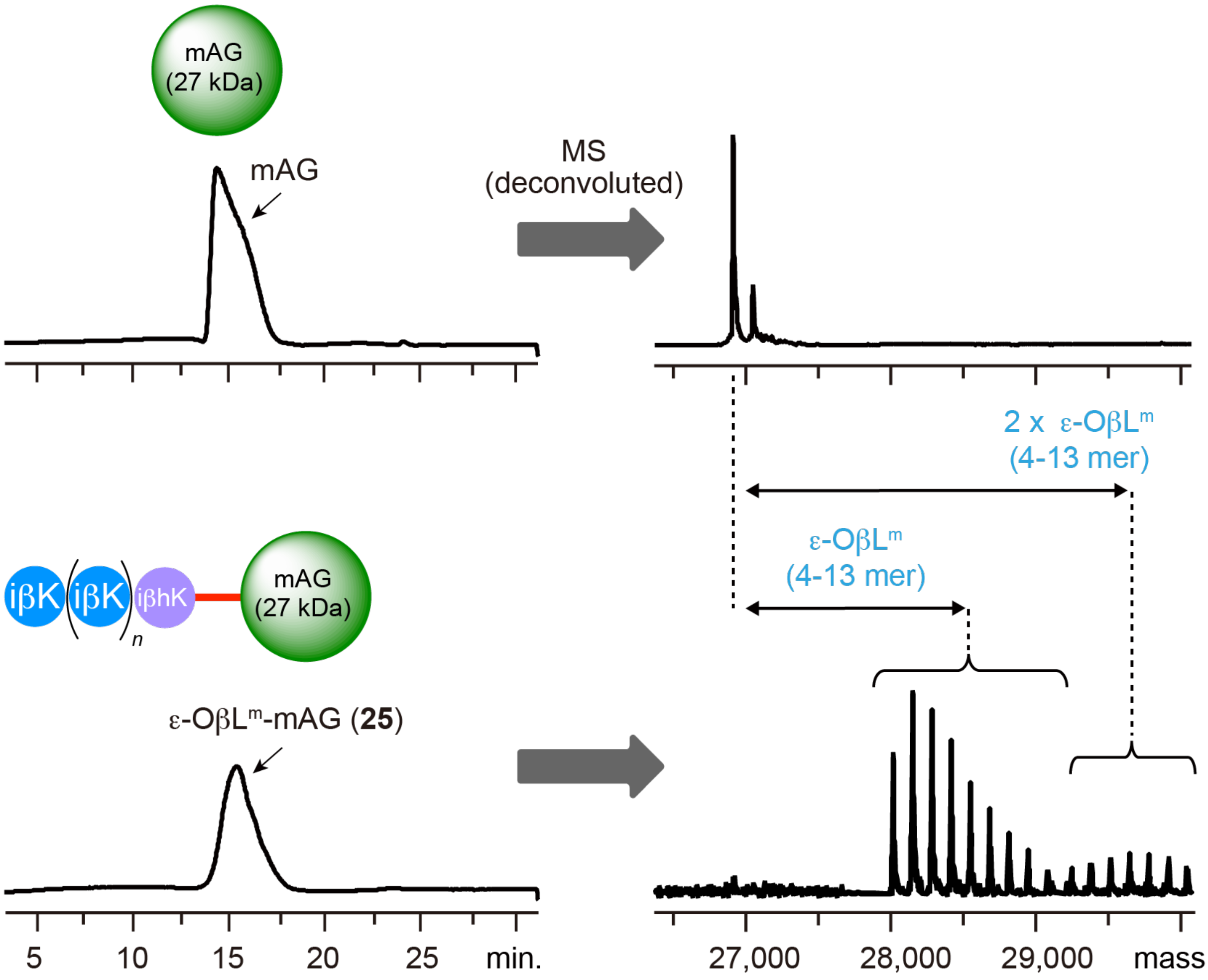

The chemically synthesized ε-OβL^{m}-mAG (**25**) consisting of 4-13 mer was analyzed by HPLC-HR-ESI-MS. The total ion current (TIC) chromatograms are shown.

(Continued on the next page)

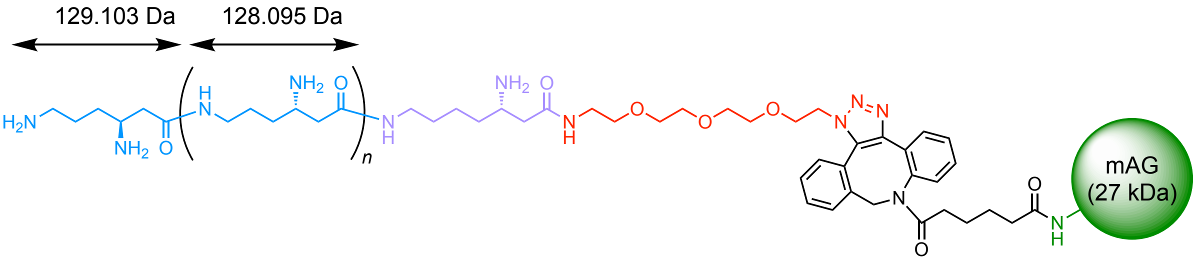

compound (**ID**)	peptide -chain length	molecular formula	calculated mass [a]	observed mass (deconvoluted) [a]	monomer-unit difference [b] (Da)	mass discrepancy [c] (Da)
mAG	N/A	$C_{1210}H_{1846}N_{328}O_{350}S_{12}$	26,972.42	26,952.06	N/A	-20.36
**25**	4 mer	$C_{1264}H_{1931}N_{341}O_{359}S_{12}$	28,032.75	28,012.25	Δ 128.90	-20.5
	5 mer	$C_{1270}H_{1943}N_{343}O_{360}S_{12}$	28,160.93	28,141.15	Δ 128.31	-19.78
	6 mer	$C_{1276}H_{1955}N_{345}O_{361}S_{12}$	28,289.10	28,269.46	Δ 128.55	-19.64
	7 mer	$C_{1282}H_{1967}N_{347}O_{362}S_{12}$	28,417.27	28,398.01	Δ 128.42	-19.26
	8 mer	$C_{1288}H_{1979}N_{349}O_{363}S_{12}$	28,545.44	28,526.43	Δ 127.62	-19.01
	9 mer	$C_{1294}H_{1991}N_{351}O_{364}S_{12}$	28,673.62	28,654.05	Δ 128.27	-19.57
	10 mer	$C_{1300}H_{2003}N_{353}O_{365}S_{12}$	28,801.79	28,782.32	Δ 128.68	-19.47
	11 mer	$C_{1306}H_{2015}N_{355}O_{366}S_{12}$	28,929.96	28,911.00	Δ 126.97	-18.96
	12 mer	$C_{1312}H_{2027}N_{357}O_{367}S_{12}$	29,058.13	29,037.97	Δ 128.54	-20.16
	13 mer	$C_{1318}H_{2039}N_{359}O_{368}S_{12}$	29,186.31	29,166.51		-19.80

[a] The average molecular masses are shown.

[b] The molecular mass of the L-βLys monomer unit is calculated to be 128.095 Da.

[c] Unexpected MS discrepancies (approximately -20 Da) between the calculated and observed MSs, suggesting the post-translation modification of the recombinant mAG during the overexpression in *E. coli*.

Supplementary Table 29

PCR primers used in this study.

oligonucleotides	sequences (5' → 3')	experiments
pET21a_mAG-F	GGATTC**CATATG**GTGAGTGTGATTAAACCAGAG	overexpression of mAG
pET21a_mAG-R	CCG**CTCGAG**CTTGGCCTGACTCGGCAGCAT	overexpression of mAG
pET21a_mKO-F	GGATTC**CATATG**GTGAGTGTGATTAAACCAGAG	overexpression of mKO
pET21a_mKO-R	CCG**CTCGAG**GGAATGAGCTACTGCATCTTC	overexpression of mKO
pET21a_Cre-F	**CATATG**TCCAATTTACTGACCGTACACC	overexpression of Cre
pET21a_Cre-R	**CTCGAG**ATCGCCATCTTCCAGCAGGCGCAC	overexpression of Cre
pmKO1_mAG-F	**GAATTC**GTGAGTGTGATTAAACCAGAG	Cre reporter plasmid
pmKO1_mAG-R	**AAGCTT**TTACTTGGCCTGACTCGGCAGCAT	Cre reporter plasmid

The restriction enzyme sites used for cloning are shown in bold.

Supplementary Table 30

Construction of the Cre activity reporter plasmid (pmKO_loxP_mAG).

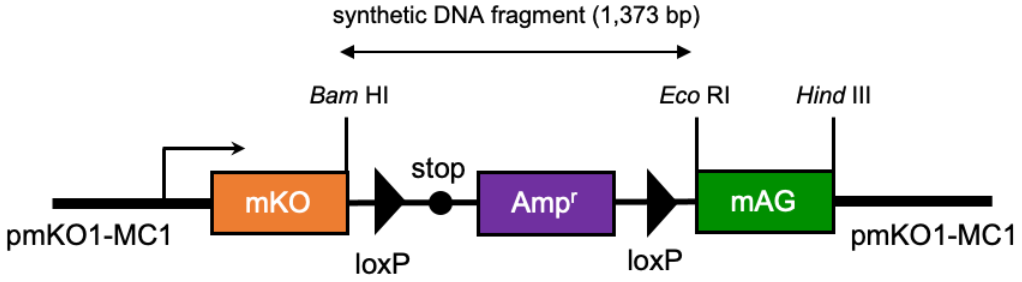

synthetic DNA sequence (1,373 bp, 5' → 3')

site/gene	start position (bp)	end position (bp)
Bam HI site	1	6
loxP site	7	40
stop codon	43	45
Amp^{r} gene	110	1,295
loxP site	1,332	1,367
Eco RI site	1,368	1,373
mAG gene	1,374	2,051
Hind III site	2,052	2,057

Bam HI loxP stop codon
GGATCCATAACTTCGTATAGCATACATTATACGAAGTTATCA**TGA**GACGAAAGGGCCTCGTGATACGCCTATTTTTATA
Amp^{r}
GGTTAATGTCATGATAATAATGGTTTCTTAGACGTCAGGTGGCACTTTTCGGGGAAATGTGCGCGGAACCCCTATTTGT
TTATTTTTCTAAATACATTCAAATATGTATCCGCTCATGAGACAATAACCCTGATAAATGCTTCAATAATATTGAAAAA
GGAAGAGTATGAGTATTCAACATTTCCGTGTCGCCCTTATTCCCTTTTTTGCGGCATTTTGCCTTCCTGTTTTTGCTCA
CCCAGAAACGCTGGTGAAAGTAAAAGATGCTGAAGATCAGTTGGGTGCACGAGTGGGTTACATCGAACTGGATCTCAAC
AGCGGTAAGATCCTTGAGAGTTTTCGCCCCGAAGAACGTTTTCCAATGATGAGCACTTTTAAAGTTCTGCTATGTGGCG
CGGTATTATCCCGTATTGACGCCGGGCAAGAGCAACTCGGTCGCCGCATACACTATTCTCAGAATGACTTGGTTGAGTA
CTCACCAGTCACAGAAAAGCATCTTACGGATGGCATGACAGTAAGAGAATTATGCAGTGCTGCCATAACCATGAGTGAT
AACACTGCGGCCAACTTACTTCTGACAACGATCGGAGGACCGAAGGAGCTAACCGCTTTTTTGCACAACATGGGGGATC
ATGTAACTCGCCTTGATCGTTGGGAACCGGAGCTGAATGAAGCCATACCAAACGACGAGCGTGACACCACGATGCCTGT
AGCAATGGCAACAACGTTGCGCAAACTATTAACTGGCGAACTACTTACTCTAGCTTCCCGGCAACAATTAATAGACTGG
ATGGAGGCGGATAAAGTTGCAGGACCACTTCTGCGCTCGGCCCTTCCGGCTGGCTGGTTTATTGCTGATAAATCTGGAG
CCGGTGAGCGTGGGTCTCGCGGTATCATTGCAGCACTGGGGCCAGATGGTAAGCCCTCCCGTATCGTAGTTATCTACAC
GACGGGGAGTCAGGCAACTATGGATGAACGAAATAGACAGATCGCTGAGATAGGTGCCTCACTGATTAAGCATTGGTAA
CTGTCAGACCAAGTTTACTCATATATACTTTAGATTGATTTAAAACTTCATTTTTAATTTAAAAGGATCTAGGTGAAGA
TCCTTTTTGATAATCTCATGACCAAAATCCCTTAACGTGAGTTTTCGTTCCACTGAGCGTCAGACCCCGTAGAAAAGAT
CAAAGGATCTTCTTGAGATCCTTTTTTTCTGCGCGTAATCTGCTGCTTGCAAACAAAAAAACCACCGATAACTTCGTAT
loxP *Eco* RI mAG
AGCATACATTATACGAAGTTATCAGAATTCGTGAGTGTGATTAAACCAGAGATGAAGATCAAGCTGTGTATGAGAGGCA
CTGTAAACGGGCATAATTTCGTGATTGAAGGAGAAGGAAAAGGAAATCCTTACGAGGGAACGCAGATTTTAGACCTGAA
CGTCACTGAAGGCGCACCTCTGCCTTTCGCTTACGATATCTTGACAACAGTGTTCCAGTACGGCAACAGGGCATTCACC
AAGTACCCAGCAGATATTCAGGACTATTTCAAGCAGACTTTTCCTGAGGGGTATCACTGGGAAAGAAGCATGACTTATG
AAGACCAGGGCATTTGCACCGCCACAAGCAACATAAGCATGAGGGGCGACTGTTTTTTCTATGACATTCGTTTTGATGG
CACCAACTTTCCTCCCAATGGTCCGGTTATGCAGAAGAAGACTCTTAAATGGGAGCCATCCACTGAGAAAATGTACGTA
GAGGATGGAGTGCTGAAGGGTGATGTTAACATGCGCCTGTTGCTTGAAGGAGGTGGCCATTATCGATGTGATTTCAAAA
CTACTTACAAAGCAAAGAAGGAGGTCCGTTTGCCAGACGCGCACAAAATTGACCACCGCATTGAGATTTTGAAGCATGA
CAAAGATTACAACAAGGTCAAGCTCTATGAGAATGCCGTTGCTCGCTATTCTATGCTGCCGAGTCAGGCCAAGTAAAAG
CTT
Hind III

3. Supplementary Note

Synthesis of L-βLys

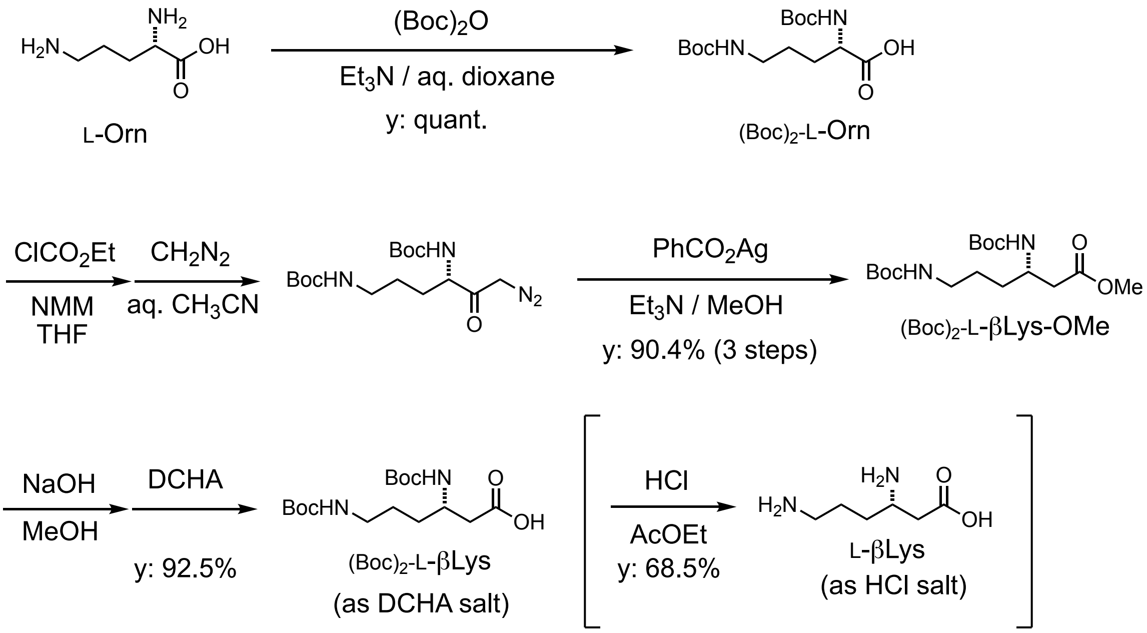

Materials: Silica-gel TLC and column chromatography were performed on TLC 60 F254 (Merck) and silica-gel 60 (neutral-type; Kanto Chemical), respectively. Air- and/or moisture-sensitive reactions were carried out under a nitrogen atmosphere with oven-dried glassware. Evaporation and concentration were carried out under reduced pressure below 30°C. All chemicals were obtained from commercial sources and used without further purification.

$(Boc)_2$-L-Orn: To a stirred solution of L-ornitine (L-Orn) hydrochloride (10.0 g, 59.8 mmol) and Et_3N (28 ml, 200 mmol) in 66% aqueous 1,4-dioxane (250 ml) was added $(Boc)_2O$ (30 g, 140 mmol) at 0°C and the mixture was stirred at room temperature overnight. After evaporation of 1,4-dioxane, the aqueous solution was washed with petroleum ether (100 ml × 3) to remove unreacted $(Boc)_2O$, adjusted to pH 3.0 with citric acid, and extracted three times with AcOEt. The combined organic layers were washed with brine and dried (Na_2SO_4). Evaporation and trituration with petroleum ether afforded $(Boc)_2$-L-Orn as a colorless power (20.5 g, quant.): ^{1}H-NMR (400 MHz, $CDCl_3$) δ (ppm) 1.45 (18H, s, (C$\underline{H}_3$)C-×2), 1.60 (m, 2H, H3), 1.69 (m, 1H, H4), 1.88 (m, 1H, H4), 3.15 (m, 2H, H5), 4.33 (m, 1H, H2), 4.84 (brs, 1H, NH), 5.30 (brs, 1H, NH), 7.80 (br, 1H, CO_2H); ^{13}C-NMR (100 MHz, $CDCl_3$) δ (ppm) 25.9 (C4), 28.3 (($\underline{C}H_3$)C-), 28.4 (($\underline{C}H_3$)C-), 29.8 (C3), 39.9 (C5), 53.0 (C2), 79.2 ((CH_3)$\underline{C}$-), 79.3 ((CH_3)$\underline{C}$-), 155.7 ($\underline{C}$ONH), 156.4 ($\underline{C}$ONH), 175.8 ($\underline{C}O_2H$).

$(Boc)_2$-L-βLys-OMe[1]: To a stirred solution of $(Boc)_2$-L-Orn (7.5 g, 22.5 mmol) and *N*-methyl morpholine (2.7 ml, 25 mmol) in dry THF (125 ml) was added ethyl chloroformate

(2.4 g, 25 mmol) at -20°C. The reaction mixture was stirred for 2 h, filtered, and washed with dry THF. At 4°C, to this was added dry CH_3CN (50 ml) and an ethereal CH_2N_2 solution (100 ml) which had been prepared by careful addition of *p*-tolylsulfonylmethylnitrosamide (21.5 g, 100 mmol) in Et_2O (175 mL) to a heated (75°C) solution of KOH (6 g) in H_2O/diethylene glycol monoethyl ether = 10/35 mL during 20 min and distillation[2], and the mixture was stirred overnight at the same temperature. After excess CH_2N_2 was removed by warming at 50°C for 1 h, the mixture was adjusted to pH 4.0 by AcOH/$CHCl_3$, filtered, evaporated, and azeotroped with toluene. The resulting crystalline dizoketone was dissolved in dry MeOH (100 ml) and $PhCO_2Ag$ (1.14 g, 5 mmol) and Et_3N (15 ml) were carefully added at room temperature. The mixture was stirred in the dark for 2 h, evaporated, suspended on AcOEt, and filtered through a diatomite pad (Radiolite #700, Showa Chem. Ind., Tokyo, Japan), which was used as a filtration aid placed on a filter paper in a Büchner funnel. The filtrate was subsequently washed with sat. $NaHCO_3$, H_2O, 10% citric acid, H_2O, and brine, and dried (Na_2SO_4). Evaporation and purification by column chromatography (silica, hexane/AcOEt = 7/3→6/4) gave $(Boc)_2$-L-β-Lys-OMe as a pale yellowish oil (7.32 g, 90.4%, 3 steps): ^{1}H-NMR (400 MHz, $CDCl_3$) δ (ppm) 1.44 (18H, s, $(C\underline{H}_3)C$-×2), 1.52 (m, 4H, H4+H5), 2.52 (m, 2H, H2), 3.12 (m, 2H, H6), 3.68 (s, 3H, $-CO_2C\underline{H}_3$), 3.90 (m, 1H, H3), 4.65 (brs, 1H, NH), 4.99 (brs, 1H, NH); ^{13}C-NMR (100 MHz, $CDCl_3$) δ (ppm) 26.8 (C5), 28.3 ($(\underline{C}H_3)C$-), 28.4 ($(\underline{C}H_3)C$-), 31.8 (C4), 39.2 (C2), 40.2 (C6), 47.3 (C3), 51.7 ($-CO_2\underline{C}H_3$), 79.1 ($(CH_3)\underline{C}$-), 79.3 ($CH_3)\underline{C}$-), 155.4 ($\underline{C}ONH$), 156.0 ($\underline{C}ONH$), 172.0 ($\underline{C}O_2CH_3$).

$(Boc)_2$-L-βLys·DCHA: To a stirred solution of $(Boc)_2$-L-βLys-OMe (7.32 g, 20.3 mmol) in MeOH (30 ml) was added 2M NaOH (25 ml) and MeOH (30 ml) at room temperature and the mixture was stirred overnight. After evaporation of MeOH, the residues were suspended in CH_2Cl_2 / 10% citric acid and extracted three times with CH_2Cl_2. The combined organic layers were washed with H_2O, and brine, and dried (Na_2SO_4). An yellow gummy product thus obtained by evaporation was mixed with dicyclohexylamine (3.79 ml, 19 mmol) and triturated with AcOEt / petroleum ether to give $(Boc)_2$-L-βLys·DCHA as a pale yellowish powder (9.90 g, 92.5%): ^{1}H-NMR (400 MHz, $CDCl_3$) δ (ppm) 1.33 (m, 4H, DCHA), 1.36 (m, 4H, DCHA), 1.44 (18H, s, $(C\underline{H}_3)C$-×2), 1.55 (m, 2H, H4), 1.65 (m, 2H, H5), 1.78 (m, 4H, DCHA), 1.80 (m, 4H, DCHA), 2.34 (dd, 1H, J = 4.0, 15.0 Hz, H2), 2.39 (dd, 1H, J = 5.0, 15.0 Hz, H2), 2.90 (t, 2H, J = 11.0 Hz, DCHA), 3.11 (m, 2H, H6), 3.75 (m, 1H, C3), 4.75 (m, 1H, DCHA), 6.33 (brs, 1H, NH), 7.87 (br, other exchangeable protons); ^{13}C-NMR (100 MHz, $CDCl_3$) δ (ppm) 24.8 (DCHA), 25.4 (DCHA), 26.7 (C5), 28.4 ($(\underline{C}H_3)C$-), 28.5 ($(\underline{C}H_3)C$-), 30.0 (DCHA), 30.1 (DCHA), 32.4 (C4), 40.5 (C2), 41.9 (C6), 47.8 (C3), 52.5 (DCHA), 78.3 ($(CH_3)\underline{C}$-), 78.8 ($CH_3)\underline{C}$-), 155.7 ($\underline{C}ONH$), 155.9 ($\underline{C}ONH$), 177.0 ($\underline{C}O_2H$).

L-βLys·HCl: $(Boc)_2$-L-βLys·DCHA (791 mg, 1.5 mmol) was acid extracted with AcOEt / 10% citric acid to remove DCHA. After evaporating the solvent, (the resulting $Boc)_2$-L-βLys was treated with 4M HCl /AcOEt (5 ml) at room temperature for 3 h. To the mixture were added $CHCl_3$ and H_2O and an aqueous layer was washed three times with $CHCl_3$, and

lyophilized. L-βLys·HCl was obtained as a green-yellowish powder (0.225 g, 68.5%): ^{1}H-NMR (400 MHz, D_2O) (relative to HOD set to 4.87) δ (ppm) 1.56 (m, 4H, H4+H5), 2.51 (dd, 1H, *J* = 8.0, 17.8 Hz, H2), 2.65 (dd, 1H, *J* = 4.5, 17.8 Hz, H2), 2.82 (m, 2H, H6), 3.47 (m, 1H, H3); ^{13}C-NMR (100 MHz, D_2O) δ (ppm) 22.8 (C5), 28.9 (C4), 35.5 (C2), 38.8 (C6), 47.7 (C3), 173.8 ($\underline{C}O_2H$).

NMR analysis: ^{1}H- and ^{13}C-NMR spectra were recorded on a Bruker AVANCE 400 spectrometer (Bruker) in $CDCl_3$ or D_2O as a solvent using TMS as an internal standard unless otherwise noted.

References

1. T. Wakamiya, H. Uratani, T. Teshima, and T. Shiba, Synthesis of acyl derivatives of β-lysine for peptide synthesis. *Bull. Chem. Soc. Jpn*. **48**, 2401-2402 (1975)
2. T.J de Boer and H.J. Backer, Diazomethane, *Org. Synth*. **36**, 16 (1956)
