# Supplementary material for: First direct evidence for direct cell-membrane penetrations of polycationic homopoly(amino acid)s produced by bacteria
Source: Commun Biol. 2022 Oct 26;5:1132. doi: 10.1038/s42003-022-04110-4 (PMC9606270; doi:10.1038/s42003-022-04110-4)
Supplement: Supplementary file 3 — Description of Additional Supplementary Data [file 42003_2022_4110_MOESM3_ESM.pdf]

Description of Additional Supplementary Files

File name: Supplementary Data 1

Description: The source data behind the graphs in Fig 3c-j.

File name: Supplementary Data 2

Description: The source data behind the graphs in Fig 4b-g.

File name: Supplementary Data 3

Description: The source data behind the graphs in Fig 8b-e.

File name: Supplementary Data 4

Description: The source data behind the graphs in Supplementary Fig 7a-f.

File name: Supplementary Data 5

Description: The source data behind the graphs in Supplementary Table 19.

File name: Supplementary Movie 1

Description: Uptake of ε-PαL-mAG

File name: Supplementary Movie 2

Description: Uptake of R8-mKO

File name: Supplementary Movie 3

Description: After uptake of ε-PαL-mAG

File name: Supplementary Movie 4

Description: After uptake of R8-mKO
