# Supplementary material for: First direct evidence for direct cell-membrane penetrations of polycationic homopoly(amino acid)s produced by bacteria
Source: Commun Biol. 2022 Oct 26;5:1132. doi: 10.1038/s42003-022-04110-4 (PMC9606270; doi:10.1038/s42003-022-04110-4)
Supplement: Supplementary file 13 — Reporting Summary-New [file 42003_2022_4110_MOESM13_ESM.pdf]

nature portfolio

Corresponding author(s): Yoshimitsu Hamano

Last updated by author(s): Oct 4, 2022

Reporting Summary

Nature Portfolio wishes to improve the reproducibility of the work that we publish. This form provides structure for consistency and transparency in reporting. For further information on Nature Portfolio policies, see our Editorial Policies and the Editorial Policy Checklist.

Statistics

For all statistical analyses, confirm that the following items are present in the figure legend, table legend, main text, or Methods section.

n/a	Confirmed	
☐	☒	The exact sample size (*n*) for each experimental group/condition, given as a discrete number and unit of measurement
☐	☒	A statement on whether measurements were taken from distinct samples or whether the same sample was measured repeatedly
☐	☒	The statistical test(s) used AND whether they are one- or two-sided<br>*Only common tests should be described solely by name; describe more complex techniques in the Methods section.*
☒	☐	A description of all covariates tested
☒	☐	A description of any assumptions or corrections, such as tests of normality and adjustment for multiple comparisons
☐	☒	A full description of the statistical parameters including central tendency (e.g. means) or other basic estimates (e.g. regression coefficient) AND variation (e.g. standard deviation) or associated estimates of uncertainty (e.g. confidence intervals)
☐	☒	For null hypothesis testing, the test statistic (e.g. *F*, *t*, *r*) with confidence intervals, effect sizes, degrees of freedom and *P* value noted<br>*Give P values as exact values whenever suitable.*
☒	☐	For Bayesian analysis, information on the choice of priors and Markov chain Monte Carlo settings
☒	☐	For hierarchical and complex designs, identification of the appropriate level for tests and full reporting of outcomes
☒	☐	Estimates of effect sizes (e.g. Cohen's *d*, Pearson's *r*), indicating how they were calculated

Our web collection on statistics for biologists contains articles on many of the points above.

Software and code

Policy information about availability of computer code

Data collection	SoftMax Pro7 software and Gen 5 (ver. 1.09) were used to export intracellular delivery experiment data from plate readers (SpectraMax iD3, Molecular device; SYNERGY 4, BioTek).<br>High-resolution mass spectra were collected by otof Control software (maXis plus; Bruker)
Data analysis	GraphPad Prism software (ver. 8; GraphPad, San Diego, CA, USA) was used for graphing and statistical analysis.<br>High-resolution mass spectra were analyzed by Compass DataAnalysis program (Bruker)

For manuscripts utilizing custom algorithms or software that are central to the research but not yet described in published literature, software must be made available to editors and reviewers. We strongly encourage code deposition in a community repository (e.g. GitHub). See the Nature Portfolio guidelines for submitting code & software for further information.

Data

Policy information about availability of data

All manuscripts must include a data availability statement. This statement should provide the following information, where applicable:

- Accession codes, unique identifiers, or web links for publicly available datasets
- A description of any restrictions on data availability
- For clinical datasets or third party data, please ensure that the statement adheres to our policy

All data is available in the main Figures and Supplementary Figures. Raw data for Figures 3c-j, 4b-g, 8b-e, Supplementary Figures 7a-f, and Supplementary table 19 are available in the source data files.

Human research participants

Policy information about studies involving human research participants and Sex and Gender in Research.

Reporting on sex and gender	n/a
Population characteristics	n/a
Recruitment	n/a
Ethics oversight	n/a

Note that full information on the approval of the study protocol must also be provided in the manuscript.

Field-specific reporting

Please select the one below that is the best fit for your research. If you are not sure, read the appropriate sections before making your selection.

☒ Life sciences ☐ Behavioural & social sciences ☐ Ecological, evolutionary & environmental sciences

For a reference copy of the document with all sections, see nature.com/documents/nr-reporting-summary-flat.pdf

Life sciences study design

All studies must disclose on these points even when the disclosure is negative.

Sample size	No statical methods were used to predetermine sample size. Where statical analyses were performed, the sample sizes are listed in the corresponding figure legends.
Data exclusions	No data was excluded.
Replication	All experiments presented in the manuscript were reproducible. Information regarding number of biological and technical replicates is reported for individual experiments in the figure legends.
Randomization	Randomization was not applicable to this study as no clinical or animal experiments were done.
Blinding	Blinding was not relevant to this study. All sample were treated equally in the intracellular delivery experiments, and all data was acquired by unbiased automated means (plate reader).

Reporting for specific materials, systems and methods

We require information from authors about some types of materials, experimental systems and methods used in many studies. Here, indicate whether each material, system or method listed is relevant to your study. If you are not sure if a list item applies to your research, read the appropriate section before selecting a response.

Materials & experimental systems

n/a	Involved in the study
☐	☒ Antibodies
☐	☒ Eukaryotic cell lines
☒	☐ Palaeontology and archaeology
☒	☐ Animals and other organisms
☒	☐ Clinical data
☒	☐ Dual use research of concern

Methods

n/a	Involved in the study
☒	☐ ChIP-seq
☒	☐ Flow cytometry
☒	☐ MRI-based neuroimaging

Antibodies

Antibodies used

Anti alpha-tubulin, monoclonal antibody (Code no., 013-25033. Lot no., CAF6686) was purchased from Fujifilm Wako Pure Chemical (Japan).

Validation

The antibody is validated by the manufacturer.
https://labchem-wako.fujifilm.com/us/product/result/product.html?fw=Anti%20alpha-tubulin,%20monoclonal%20antibody

Eukaryotic cell lines

Policy information about cell lines and Sex and Gender in Research

Cell line source(s)

HeLa: RIKEN BioResource Research Center (Japan)
HEK293T: RIKEN BioResource Research Center (Japan)
HepG2: RIKEN BioResource Research Center (Japan)
K562: RIKEN BioResource Research Center (Japan)
SH-SY5Y: ATCC (USA)
HDF: PromCell (Germany)

Authentication

We purchased the cell lines (HeLa, HEK293T, HepG2, K562) authenticated by RIKEN BioResource Research Center (Japan) were not further validated. The cell lines, SH-SY5Y and HDF, were not authenticated.

Mycoplasma contamination

We purchased the cell lines (HeLa, HEK293T, HepG2, K562) tested by RIKEN BioResource Research Center (Japan), and they were not further tested for mycoplasma contamination. The cell lines, SH-SY5Y and HDF, were not tested.

Commonly misidentified lines
(See ICLAC register)

No commonly misidentified cell lines were not used.
